# Multi-omics prediction of axillary treatment response and tumour microenvironment alterations in lymph node-positive luminal breast cancer

**DOI:** 10.1038/s41419-025-07877-6

**Published:** 2025-08-04

**Authors:** Teng Ma, Zirui Wang, Zhe Zhou, Yongmei Wang, Tianyi Ma, Xiangping Liu, Yan Mao, Haibo Wang

**Affiliations:** 1 Breast Disease Center, Affiliated Hospital of Qingdao University, Qingdao, 266000 China; 2 Zhongtai Securities Institute for Financial Studies, Shandong University, Jinan, 250100 China; 3 Department of Radiology, Affiliated Hospital of Jining Medical University, Jining, 272100 China; 4 Medical Research Center, Affiliated Hospital of Qingdao University, Qingdao, 266000 China

**Keywords:** Breast cancer, Translational research

## Abstract

Luminal breast cancer (BC) with axillary lymph node (ALN) metastasis is typically treated with neoadjuvant chemotherapy (NAC). Theoretically, patients who achieve pathological lymph node complete response after NAC can be exempted from ALN dissection and even have the possibility of being spared axillary surgery. However, there is no effective way to preoperatively assess whether a metastatic ALN achieved pathological lymph node complete response (pLCR) after NAC. Therefore, we retrospectively collected imaging, clinical, and pathological data from two centres, built a multi-omic model to predict pLCR, and validated its accuracy and clinical applicability. We identified 12 radiomic and four clinicopathological features for model construction; the areas under the curve for training and validation cohorts were 0.853 and 0.805, respectively. Subsequently, single-cell RNA sequencing analysis was conducted on patients with different efficacy and its association with the tumour immune microenvironment was investigated. Eleven cell clusters in 14 samples from five patients were identified with differing NAC responses; comparative analysis indicated that those with poor responses had immunosuppressive features, which provided a theoretical basis for elucidating the resistance mechanism of NAC in axillary metastatic lymph nodes. The multi-omics prediction model demonstrated good performance in predicting ALN status after NAC, offering the possibility of reducing unnecessary axillary surgery.

## Introduction

Breast cancer (BC) is the most common malignant tumour in women. Luminal BC (hormone receptor positive [HR+]/human epidermal growth factor receptor 2 negative [HER2−]) accounts for two-thirds of all BC [1]. Axillary lymph node (ALN) metastasis influences patient prognosis and survival [2], and neoadjuvant chemotherapy (NAC) is the first line treatment for luminal BC with ALN metastases [3, 4]. After systematic NAC, the primary breast lesion and ALN metastases can be completely resected, defined as a pathological complete response (pCR) [5, 6]. In luminal BC, the probability of lymph node pCR (pLCR) is higher than breast pCR (pBCR), and pLCR has a greater impact on disease-free and overall survival [7, 8].

Patients who achieve pLCR after NAC can be exempted from ALN dissection (ALND) and sentinel lymph node biopsy (SLNB) [9, 10]. However, the gold standard for evaluating pLCR requires histopathology of post-NAC specimens, creating a time lag. Therefore, a method for preoperative assessment that can objectively and reliably predict pLCR in these patients is needed.

Magnetic resonance imaging (MRI) is widely used to assess breast tumours and axillary status, and dynamic contrast-enhanced (DCE) MRI is reliable for tracking residual tumours following NAC [11]. Super-resolution (SR) technology was proposed in the 1980s to obtain images with higher spatial resolution for more accurate medical diagnoses [12, 13]. Radiomics is an emerging field combining medical imaging with clinical research; it extracts a large amount of quantitative information from medical images and has great potential in guiding clinical diagnoses and treatment [14–17]. This study aimed to establish a multi-omic prediction model using DCE-MRI features, clinicopathological characteristics, and machine-learning algorithms to predict the response of metastatic ALNs to NAC in luminal BC. The purpose of this model was to screen patients for pLCR, reducing unnecessary SLNB or ALND, minimising postoperative complications, and improving quality of life. Single-cell RNA sequencing (scRNA-seq) was then used to investigate tumour immune microenvironment (TIME) heterogeneity in patients with differing axillary responses following NAC.

## Results

### Baseline characteristics

**Fig. 1:**
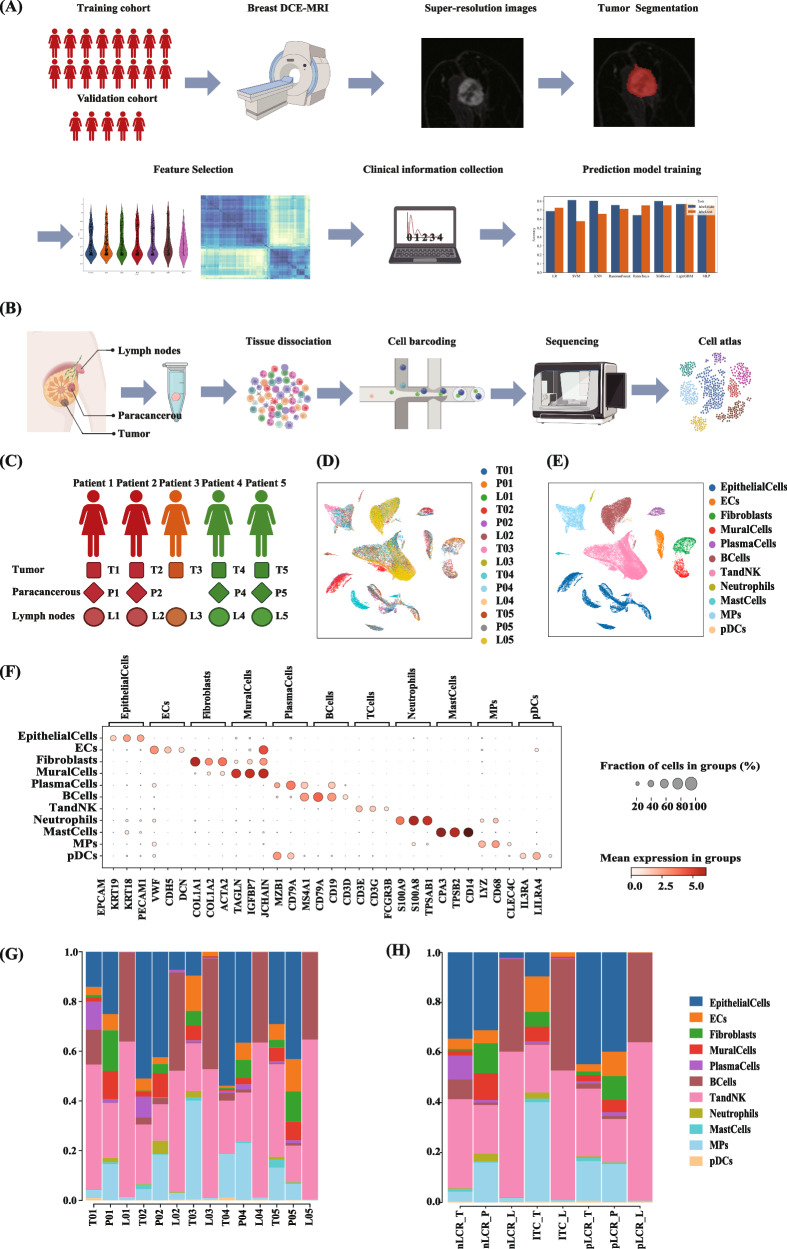
Study design and workflow. **A** Predictive model construction workflow. DCE-MRI images were retrospectively collected and enhanced by super-resolution reconstruction; tumor segmentation and image feature screening were performed, followed by downsizing the extracted features. Lastly, clinical and pathological features were combined to construct the multimodal predictive model. **B** Single-cell RNA sequencing workflow. Tissues were collected and processed for cell dissociation; these were then used to prepare single-cell RNA libraries, which were sequenced for data analysis. (C). Single-cell transcriptome sequencing sample distribution map. **D**, **E** Uniform manifold approximation and projection plot of all 86,279 cells, coloured by sample (**D**) and major cell clusters (**E**). **F** Dot plot showing the expression levels of genes that are typical markers of the major cell types. **G**, **H** The proportions of major cell clusters by sample (**G**) and subgroup (**H**). DCE-MRI dynamic contrast-enhanced magnetic resonance imaging, ECs endothelial cells, MPs mononuclear phagocytes, NK natural killer, pDCs plasmacytoid dendritic cells.

This study included 457 individuals, with 375 in the training cohort and 82 in the validation cohort. A total of 98 achieved pLCR in the training cohort (26.13%), 22 achieved pLCR in the validation cohort (26.83%). The clinical and pathological characteristics of the patients are shown in Table 1, and the study flowchart is illustrated in Fig. 1A, B. In the training cohort, the clinical N stage, histological grading, number of tumour-infiltrating lymphocytes (TILs), and presence of radiological complete responses in both the breast (rBCR) and ALNs (rLCR) were different between the pLCR and non-pLCR (nLCR) groups (*P* < 0.05). The only statistically significant difference between patients in the pLCR and nLCR groups in the validation cohort was the clinical N stage (*P* < 0.05).

**Table 1:** Clinical and pathological characteristics in the training and validation cohorts.

	Training (*n* = 375)			Validation (*n* = 82)		
	pLCR (*n* = 98)	nLCR (*n* = 277)	*P* value	pLCR (*n* = 22)	nLCR (*n* = 60)	*P* value
Age (years), no. (%)			0.382			0.722
≥35	94 (95.92)	259 (93.50)		21 (95.45)	56 (93.33)	
>35	4 (4.08)	18 (6.50)		1 (4.55)	4 (6.67)	
Menopause status, no. (%)			0.248			0.591
Premenopausal	48 (48.98)	117 (42.24)		11 (50.00)	26 (43.33)	
Postmenopausal	50 (51.02)	160 (57.76)		11 (50.00)	34 (56.67)	
Pathology, no. (%)			0.055			0.390
IDC	84 (85.71)	212 (76.53)		20 (90.91)	50 (83.33)	
Others	1414.29 ()	65 (23.47)		2 (9.09)	10 (16.67)	
T stage, no. (%)			0.122			0.147
T1	60 (61.22)	142 (51.26)		7 (31.82)	32 (53.33)	
T2	29 (29.59)	89 (32.13)		10 (45.45)	22 (36.67)	
T3	9 (9.18)	46 (16.61)		5 (22.73)	6 (10.00)	
N stage, no. (%)			0.000			0.011
N1	88 (89.80)	134 (48.38)		18 (81.82)	27 (45.00)	
N2	5 (5.10)	108 (38.99)		2 (9.09)	21 (35.00)	
N3	5 (5.10)	35 (12.64)		2 (9.09)	12 (20.00)	
Grade, no. (%)			0.037			0.118
II	83 (84.69)	206 (74.37)		21 (95.45)	49 (81.67)	
III	15 (15.31)	71 (25.63)		1 (4.55)	11 (18.33)	
Ki-67, no. (%)			0.206			0.591
≥20	53 (54.08)	170 (61.37)		11 (50.00)	34 (56.67)	
<20	45 (45.92)	107 (38.63)		11 (50.00)	26 (43.33)	
TILs, no. (%)			0.001			0.739
≥20	34 (34.69)	51 (18.41)		3 (13.64)	10 (16.67)	
<20	64 (65.31)	226 (81.59)		19 (86.36)	50 (83.33)	
rBCR, no. (%)			0.000			0.493
Presence	86 (87.76)	267 (96.39)		20 (90.91)	57 (95.00)	
Absence	12 (12.24)	10 (3.61)		2 (9.09)	3 (5.00)	
rLCR, no. (%)			0.000			0.058
Presence	78 (79.59)	257 (92.78)		18 (81.82)	57 (95.00)	
Absence	20 (20.41)	20 (7.22)		4 (18.18)	3 (5.00)	

*IDC* invasive breast cancer, *Ki-67* proliferation marker protein Ki-67, *rBCR* radiological breast complete response, *rLCR* radiological lymph node complete response, *TILs* tumour infiltrating lymphocytes.

### Single-cell sequencing and cell type identification

After quality control, 86,279 cells were analysed. Dimensionality reduction and clustering were performed using Scanpy to identify major cell clusters with similar expression patterns. Following that, each cell was categorised into one of 11 cell clusters (Fig. 1D, E) and annotated based on classical markers: epithelial cells (gene markers: *EPCAM*, *KRT19*, and *KRT18*), endothelial cells (gene markers: *PECAM1*, *VWF*, and *CDH5*), fibroblasts (gene markers: *DCN*, *COL1A1*, and *COL1A2*), mural cells (gene markers: *ACTA2*, *TAGLN*, and *IGFBP7*), plasma cells (gene markers: *JCHAIN*, *MZB1*, and *CD79A*), B cells (gene markers: *CD79A*, *MS4A1*, and *CD19*), T and natural killer (NK) cells (gene markers: *CD3D*, *CD3E*, and *CD3G*), neutrophils (gene markers: *FCGR3 B*, *S100A9*, and *S100A8*), mast cells (gene markers: *TPSAB1*, *CPA3*, and *TPSB2*), mononuclear phagocytes (MPs) (gene markers: *CD14*, *LYZ*, and *CD68*), and plasmacytoid dendritic cells (gene markers: *CLEC4C*, *IL3RA*, and *LILRA4*) (Fig. 1F). The relative abundance of the 11 major cell types is shown in Fig. 1G, H. We presented the distribution of T and NK cells, mononuclear phagocytes and fibroblasts of all samples in the Supplementary Fig. 1.

### Multi-omic prediction model construction

#### Radiomics feature extraction and selection

Using SR, the pixel volume was transformed from 1 × 1 × 1 mm^3^ to 1 × 1 × 0.25 mm^3^ while maintaining the size of the original image (Supplementary Fig. 2). A total of 1196 radiomics features (RFs) were extracted from the processed images to quantify internal tumour heterogeneity. The Mann–Whitney *U* test was used to identify 518 RFs with a correlation of *P* < 0.05. We subsequently retained 95 RFs with a correlation >0.9 between any two. Lastly, we used LASSO regression to identify 12 RFs with non-zero coefficients, and the best λ value used for the next modelling step was 0.0222. Ultimately, 12 RFs were used for subsequent modelling. Supplementary Figs. 3 and 4 show the specific RFs and their proportions.

#### Multi-omic prediction model establishment

**Fig. 2:**
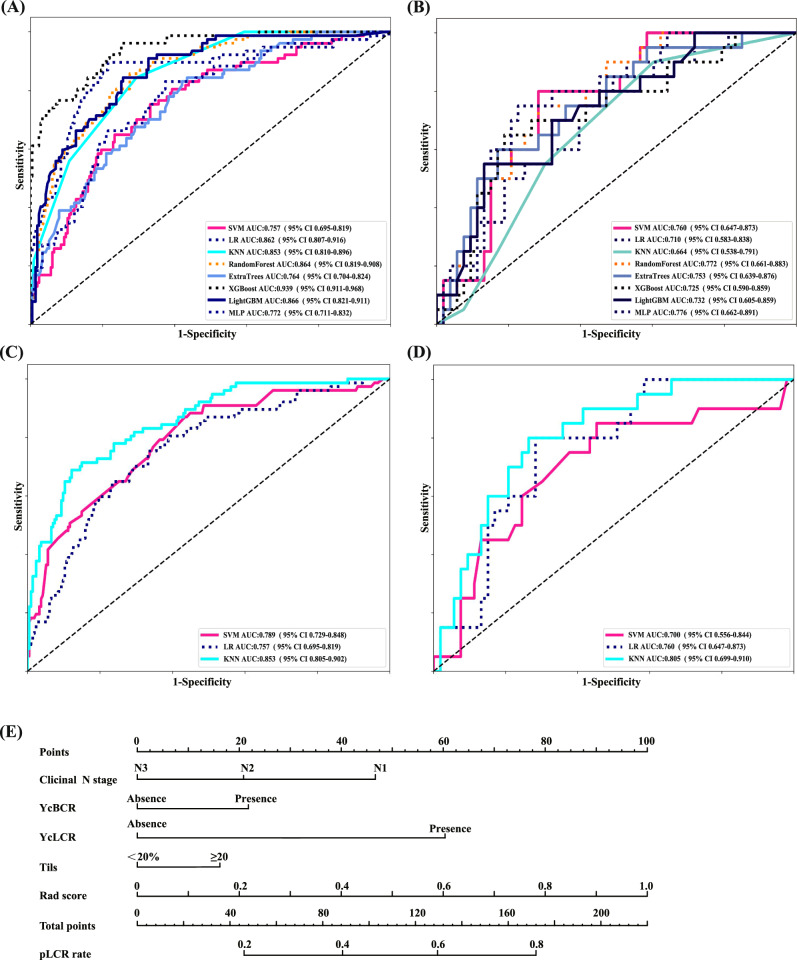
Predictive performance of the different models in the training and validation cohorts. **A**, **B** ROC curve of the radiomics prediction model based on eight machine learning algorithms (LR logistic regression, SVM support vector machine, KNN k-nearest neighbours, RF random forest, ExtraTrees extremely randomised trees, XGBoost extreme gradient boosting, LightGBM light gradient boosting machine, MLP multi-layer perception) in the training (**A**) and validation (**B**) cohorts. **C**, **D** ROC curve of the multimodal prediction model based on the SVM algorithm in the training (**C**) and validation (**D**) cohorts. **E** Comprehensive nomogram for predicting pLCR in patients with luminal breast cancer and axillary lymph node metastases. pLCR lymph node pathological complete response, rad-score radiomics score, ROC receiver operating characteristic, ycBCR post-neoadjuvant therapy preoperative breast complete response, ycLCR post-neoadjuvant therapy preoperative lymph node complete response.

We implemented eight machine-learning algorithms to construct the radiomic models and calculated the accuracy, area under the curve (AUC), sensitivity, and specificity of each model in both the training and validation cohorts (Fig. 2A, B; Supplementary Table 2). The support vector machine (SVM)-based model had superior performance compared with the other models. Therefore, we selected the SVM model for the construction of the multi-omic prediction model to calculate the corresponding rad-score.

Using univariate and multivariate analyses, we identified the N stage, TILs, rBCR, and rLCR as clinical predictors of pLCR after NAC (Supplementary Table 3). These four factors were combined with the SVM-based radiomic model to construct a multi-omic prediction model (Fig. 2E).

The results confirmed that the multi-omic model had better recognition ability than the unimodal radiomic and unimodal clinical prediction models (Fig. 2C, D). When the predictions were compared with the actual outcomes, the calibration curves of both the training and validation cohorts showed good consistency, and the clinical decision curve showed measurable clinical benefits (Supplementary Fig. 5).

### TIME heterogeneity in patients with differing ALN responses to NAC

#### Heterogeneity of T and NK cells

**Fig. 3:**
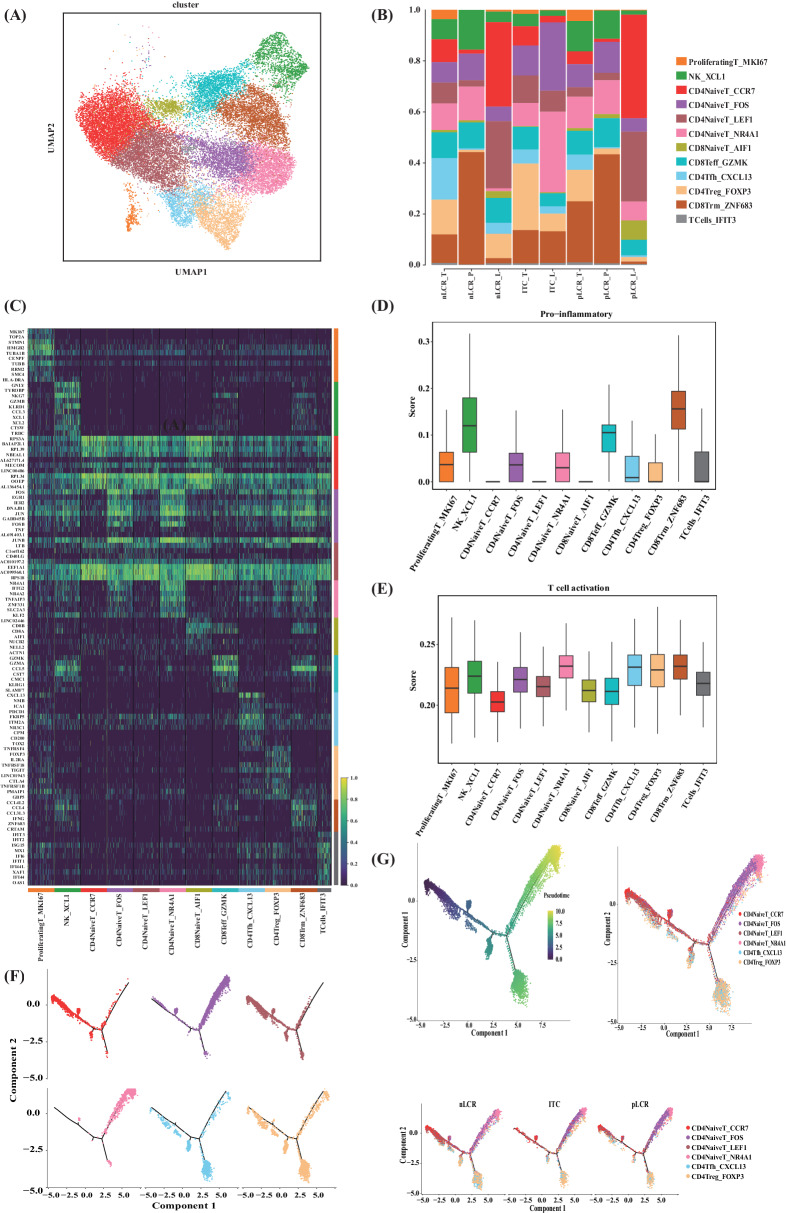
Reclustering of T and NK cells isolated from 14 samples from five patients with differing axillary responses after NAC (part 1). **A** UMAP plot of the T and NK cell landscape, coloured by subcluster. **B** The proportion of T and NK cell subclusters by sample subgroups. Subgroups were categorised based on the presence or absence of a pathological complete response in the axillary lymph nodes (pLCR and nLCR, respectively) and tissue type (tumour [T], paracancerous [P], and axillary lymph node [L]). One patient only had isolated tumour cells in the lymph node samples and was categorised separately (ITC). **C** Heatmap of the expression levels of the top 10 differentially expressed genes in the 12 T and NK cell subclusters. **D** Distribution of pro-inflammatory scores for different subclusters of T and NK cells. **E** Distribution of T cell activation signature scores of different subclusters of T and NK cells. **F**, **G** Monocle pseudotime trajectory analysis of CD4NaiveT_CCR7, CD4NaiveT_LEF1, CD4NaiveT_FOS, and CD4NaiveT_NR4A1. NAC neoadjuvant chemotherapy, NK natural killer, UMAP uniform manifold approximation and projection.

Of the immune cell types found in the samples, T and NK cells were the most abundant. To better understand their diversity and the potential variations in different patients, we performed an unsupervised clustering analysis on T and NK cells, categorising these into 12 cell subclusters (Fig. 3A, B). Of these, CD4NaiveT cells were reclassified into four cell subclusters: CD4NaiveT_FOS and CD4NaiveT_NR4A1, which had similar gene expression characteristics, and CD4NaiveT_CCR7 and CD4NaiveT_LEF1, which also had similar gene expression characteristics (Fig. 3C).

**Fig. 4:**
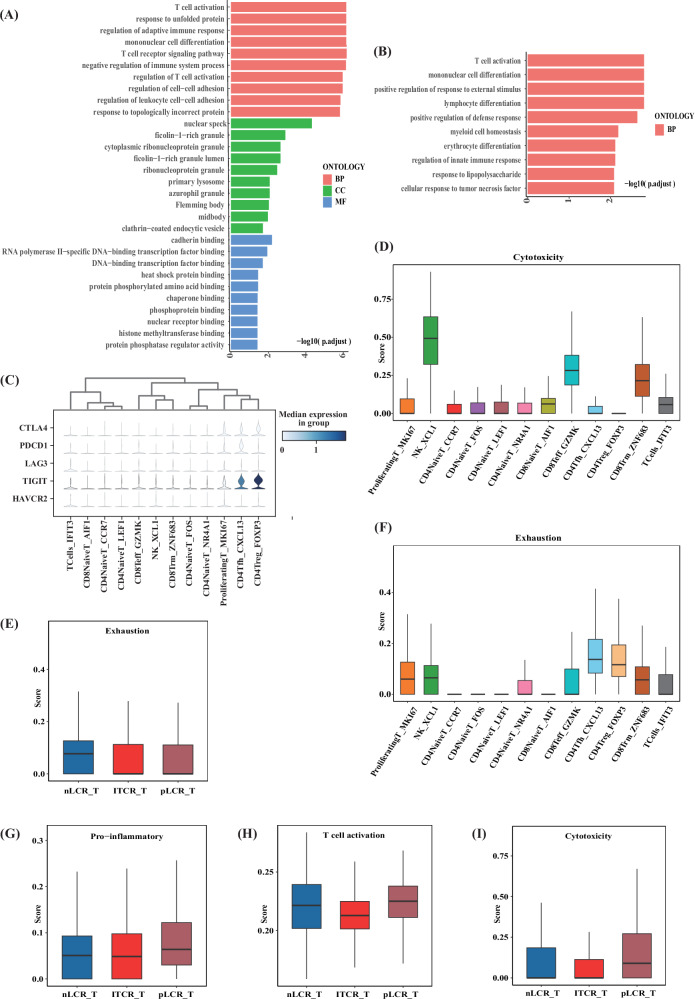
Reclustering of T and NK cells identified in 14 samples from 5 patients with differing axillary responses after NAC (part 2). **A**, **B** GO enrichment analysis showed that the CD4NaiveT_NR4A1 and CD4NaiveT_FOS subclusters in the pLCR_T group upregulated multiple immune-related pathways, including T cell activation, lymphocyte differentiation, and response to tumour necrosis factor. **C** Expression levels of typical marker genes in the subclusters. **D** Cytotoxicity scores of different subclusters of T and NK cells. **E** Differences in exhaustion scores between the subgroups. **F** Exhaustion scores of different subclusters of T and NK cells. **G** Differences in pro-inflammatory scores between the subgroups. **H** Differences in T cell activation scores between the subgroups. **I** Differences in cytotoxicity scores between the subgroups. BP biological process, CC cellular component, GO Gene Ontology, MF molecular function, NAC neoadjuvant chemotherapy, NK natural killer.

Next, we analysed subclusters with specific characteristics. The CD4NaiveT_FOS and CD4NaiveT_NR4A1 expressed multiple immediate early genes, such as *FOS*, *EGR1*, *JUN*, *IER2*, *NR4A1*, and *NR4A2* at high levels. Gene set analysis showed that the T cells in the CD4NaiveT_FOS and CD4NaiveT_NR4A1 groups concurrently had T-cell activation characteristics and pro-inflammatory scores that were higher than those of the other two groups, indicating that these were in an activated state (Fig. 3D, E). The monocle trajectory analysis confirmed this activated cell state; i.e, CD4NaiveT_CCR7 and CD4NaiveT_LEF1 were at the beginning of the developmental trajectory, and CD4NaiveT_FOS and CD4NaiveT_NR4A1 were at a later branch of development, indicating the existence of two different types of CD4NaiveT cells (Fig. 3F, G). The higher proportion of unactivated CD4NaiveT cells in the nLCR and isolated tumour cell (ITC) groups indicated that more of these cells were present in the TIME of tissues that had not reached pLCR, whereas more activated CD4NaiveT cells were found in the pLCR group. Analysis of differentially expressed genes (DEGs) using GO enrichment between the groups further demonstrated that the CD4NaiveT_FOS and CD4NaiveT_NR4A1 in the pLCR_T subgroup had upregulated multiple immune-related pathways, including T-cell activation, regulation of adaptive immune response, and response to tumour necrosis factor (Fig. 4A, B). In summary, a lack of effective CD4NaiveT cell activation may be observed in BCs that do not achieve pLCR after NAC.

CD4Tfh_CXCL13 and CD4Treg_FOXP3 were also identified among the CD4T cells and expressed high levels of the immunosuppressive molecule TIGIT (Fig. 4C). Gene pooling analyses showed high depletion signature score for these two cell types. Of these, CD4Tfh_CXCL13 cells were more common in the nLCR group, whereas CD4Treg_FOXP3 were more common in the ITC group, suggesting that more pronounced immunosuppression may be found in BCs that do not reach pLCR after NAC.

**Fig. 5:**
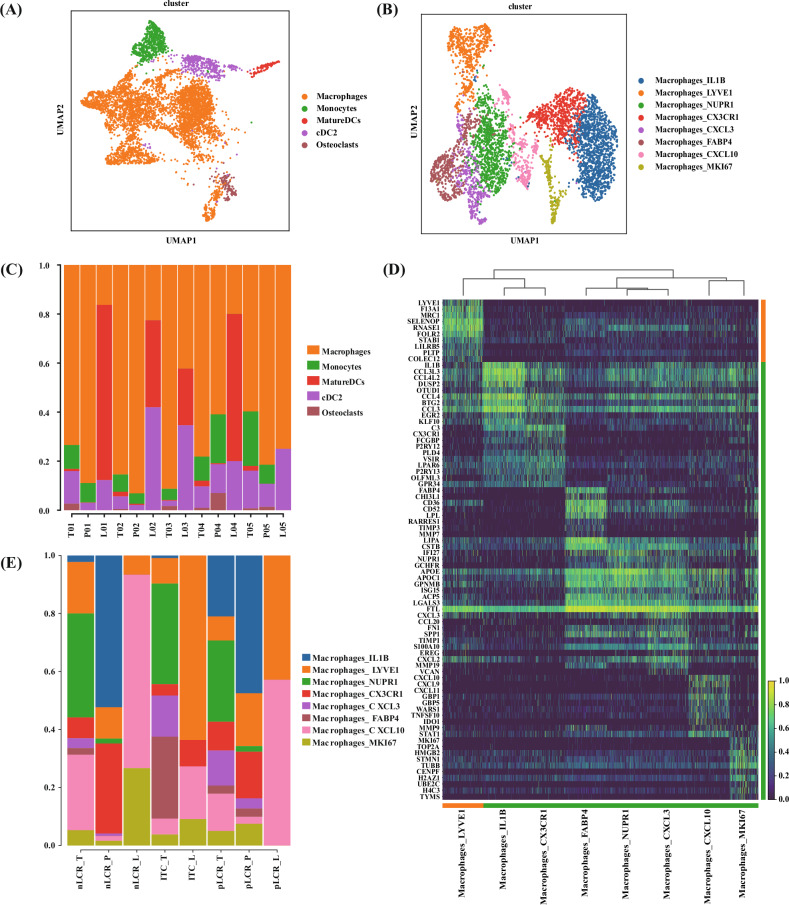
Reclustering of macrophages isolated from 14 samples from five patients with differing axillary responses after NAC (part 1). **A** UMAP plot of the mononuclear phagocyte landscape, coloured by subcluster. **B** Proportion of mononuclear phagocyte subclusters by sample. **C** Proportion of macrophage subclusters by sample subgroup. Samples were categorized based on the presence or absence of a pathological complete response in the axillary lymph nodes (pLCR and nLCR, respectively) and tissue type (tumor [T], paracancerous [P], and axillary lymph node [L]). One patient only had isolated tumor cells in the lymph node samples and was categorized separately (ITC). **D** UMAP plot of the macrophages landscape, coloured by subcluster. **E** Heatmap of the expression levels of the top ten differentially expressed genes in the eight macrophage subclusters. NAC neoadjuvant chemotherapy, UMAP uniform manifold approximation and projection.

CD8Trm_ZNF683 was more highly represented in the pLCR_T subgroup than in the other subgroups (Fig. 3B). CD8Trm_ZNF683 expressed high levels of the interferon (*IFN*)-*γ* gene, which can play a crucial part in anti-tumour responses by improving antigen presentation and stimulating effector immune cells (Fig. 3D). Furthermore, gene set analysis indicated that the CD8Trm_ZNF683 had high cytotoxicity and pro-inflammatory scores, reflecting its anti-tumour characterisation (Figs. 3C and 5D).

Differential between-group characterisation of T cells showed that the nLCR_T subgroup had the highest exhaustion score and the strongest suppressive characteristics in the TIME. The ITC_T subgroup had the weakest T-cell activation characteristics, and the T cells were potentially weakly anti-tumourigenic. The pLCR_T group had the highest cytotoxicity, activation, and pro-inflammatory characteristics, as well as the lowest exhaustion characteristics and the strongest anti-tumourigenic characteristics (Fig. 4E–I). In summary, compared with patients who achieved pLCR, those who did not had a more immunosuppressive profile and fewer T cells with an activated and cytotoxic phenotype, which may explain the poor efficacy of NAC in these individuals.

#### Heterogeneity of MPs

MPs were identified as a major cell type in the TIME and were classified into five cell subclusters, with predominantly macrophages (Fig. 5A, B). The macrophages were further subdivided into eight subclusters. The nLCR_T subgroup was relatively enriched in macrophages_LYVE1 and macrophages_CXCL10, whereas the CXCL3 subpopulation was less prominent (Fig. 5C, D).

**Fig. 6:**
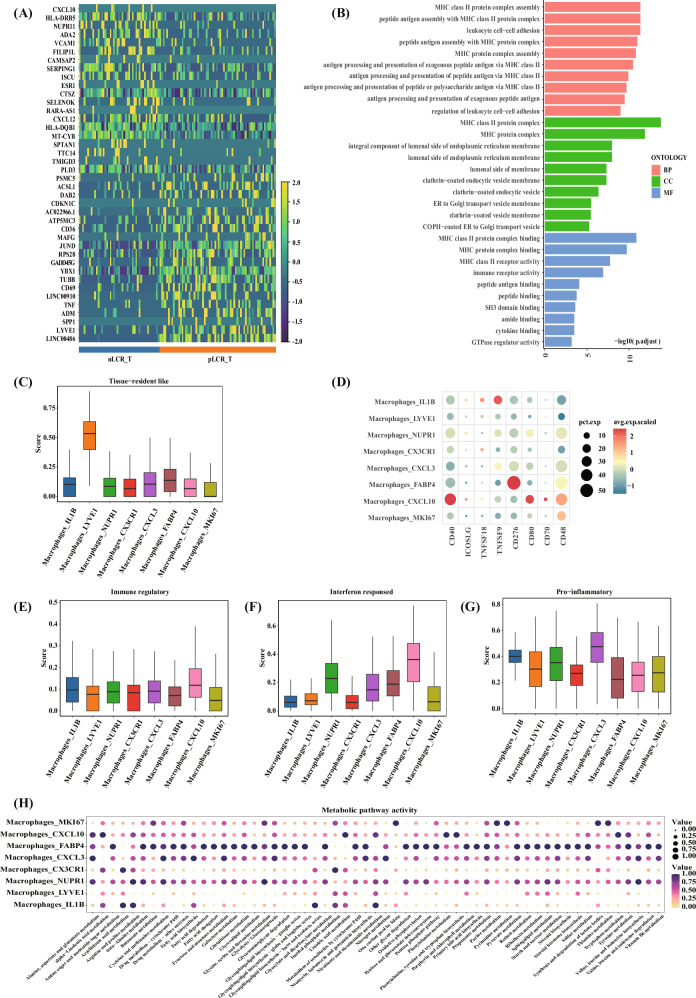
Reclustering of macrophages identified in 14 samples from five patients with differing axillary responses after NAC (part 2). **A** Tissue-resident scores of different subclusters of macrophages. **B** Differentially expressed genes between the LYVE1 subclusters. The subcluster isolated from the tumor tissues of the group who did not achieve a lymph node pathological complete response (nLCR-T) had upregulated MHCII molecules compared with the group who did achieve a complete response (pLCR-T). **C** GO enrichment analysis of the LYVE1 subcluster function. Compared with the pLCR_T subgroup, the MHCII molecules in the nLCR_T subgroup were enriched in the antigen processing and presentation pathway. **D** Expression of co-stimulatory signals in different macrophage subclusters. **E** Immune regulatory scores of different subclusters of macrophages. **F** Interferon response scores of different subclusters of macrophages. **G** Dot plot showing the ScMetabolism pathway analysis of subclusters of macrophages. **H** Pro-inflammatory scores of different subclusters of macrophages. BP biological process, CC cellular component, GO Gene Ontology, MF molecular function, NAC neoadjuvant chemotherapy.

Macrophages_LYVE1 expressed high levels of the M2 macrophage signature genes *MRC1* and *F13A1*, favouring an immunosuppressive M2 phenotype, which suggested a potentially suppressive TIME in the nLCR_T tissues (Fig. 5E). In addition, macrophages_LYVE1 expressed *FOLR2* at high levels, and this subcluster had a high tissue-resident signature score (Fig. 6A), indicating a tissue-resident macrophage subtype. Macrophages_LYVE1 also expressed high levels of *LYVE1*, *RNASE1*, *STAB1*, *CD163*, and *MARCO*, which are associated with macrophage function and tumour immunosuppression (Fig. 5E). The GO enrichment analysis demonstrated that, compared with the pLCR_T subgroup, MHCII molecules (HLA-DRB5, HLA-DQB1, among others) were upregulated in the macrophages_LYVE1 subcluster in the nLCR_T subgroup (Fig. 6B). The analysis also showed enrichment in antigen processing and presentation pathways via MHCII molecules (Fig. 6C). MHCII molecules can activate CD4 T cells; however, our findings demonstrated that more inactivated CD4-naive T cells were observed in the nLCR group. Therefore, we further analysed the expression of several costimulatory signals in the nLCR group, revealing that several costimulatory molecules were hardly expressed in macrophages_LYVE1 (Fig. 6D). Therefore, although MHCII molecules were upregulated, CD4 T cells may not have been effectively activated due to the lack of sufficient costimulatory signals.

The macrophage_CXCL10 subcluster expressed high levels of chemokine family-related genes *CXCL10*, *CXCL9*, and *CXCL11*, which are mainly involved in the recruitment of CXCR3+ T cells. Gene set analysis indicated that this subcluster was characterised by a high IFN response (Fig. 6E, F). Furthermore, ScMetabolism pathway analysis demonstrated that the macrophage_CXCL10 subcluster had higher tryptophan metabolism levels than other subclusters (Fig. 6G). These findings further suggest an immunosuppressive environment.

A special subcluster of macrophages, macrophage_CXCL3, had the highest pro-inflammatory score (Fig. 6H). The macrophage_CXCL3 subcluster accounted for the smallest proportion in the nLCR group and approximately the same proportion in the ITC and pLCR groups, which indicated that the immune activity in the nLCR group was low. In summary, a certain degree of macrophage-related immunosuppression may be present in the nLCR group, reducing anti-tumour responses. This was in line with the findings of the T-cell analysis.

### Multiplex immunofluorescence validation of the immunosuppressive microenvironment in nLCR tissues

**Fig. 7:**
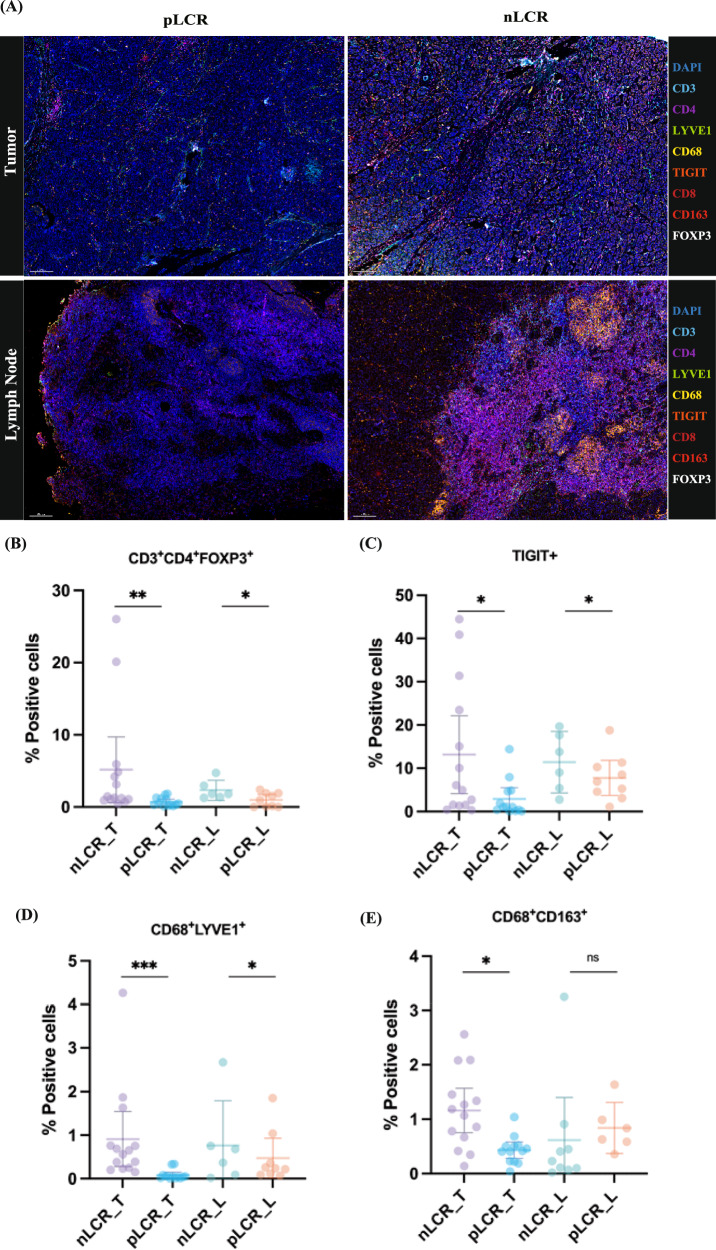
Validation of the immune microenvironment in patients with differing axillary responses using multiplex immunofluorescence. **A** Representative multiplex immunofluorescence staining images of tumor (top) and lymph node (bottom) tissues in the nLCR and pLCR groups. Scale bar = 100 μm. **B**–**E** Quantification of the percentage of CD3+ CD4+ FOXP3+ T cells (**B**), TIGIT+ cells (**C**), CD68+ LYVE1+ macrophages (**D**), and CD68+ CD163+ macrophages (**E**) among different subgroups. Data are presented as mean ± SD. *P < 0.05, **P < 0.01, ***P < 0.001.

To validate the immunosuppressive microenvironment features revealed by scRNA-seq analysis, we employed multiplex immunofluorescence on tissue samples from the different response groups (Fig. 7A). The results confirmed that, compared to the pLCR group, the tumor and lymph node tissues of the nLCR group had significantly higher infiltration of regulatory T cells (CD3+ CD4+ FOXP3+) (Fig. 7B). Furthermore, consistent with our scRNA-seq data, the proportion of cells expressing the immunosuppressive molecule TIGIT was also significantly higher in the nLCR group (Fig. 7C), which aligns with the high TIGIT expression in the CD4Tfh_CXCL13 and CD4Treg_FOXP3 subclusters. Regarding macrophages, the immunofluorescence results showed a significant increase in the number of M2-type macrophages (CD68+ LYVE1+ and CD68+ CD163+) in the tumor tissues of the nLCR group (Fig. 7D, E), validating the enrichment of immunosuppressive macrophage subclusters (such as macrophages_LYVE1) in poor-response tissues found in the scRNA-seq analysis. These protein-level validation results provide strong support for our transcriptomic findings, confirming the presence of a significant immunosuppressive tumor microenvironment in patients with poor NAC outcomes.

## Discussion

NAC has become the standard treatment for luminal BC with ALN metastases. The pCR is recognized as an important predictor of BC prognosis [5]; pCR can be divided into pLCR and pBCR [18]. At present, physical examination, ultrasound, mammography and breast MRI are used to evaluate ALN status after NAC [11]. However, each of these assessment methods have a certain false positive rate, and the pathological results of post-NAC surgical specimens continue to be the gold standard for pLCR assessment [19]. Therefore, for patients with ALN metastases, treatment of ALNs that achieve clinical negativity (ycN0) after NAC remains very cautious. For example, SLNB, ALND, and targeted axillary dissection (TAD) are all used in clinical practice [20–22]. In China, ALND after NAC is the standard procedure for most patients [23], whereas clinical guidelines in the United States and Germany recommend TAD and ALND for patients with clinically negative (ycN0) ALNs after NAC [4]. Likewise, European guidelines recommend ALND, SLNB, TLNB, and TAD for these patients [24, 25]. However, NAC can lead to lymph node structural damage, fibrosis, and potential changes in lymphatic reflux pathways. This can affect the migration of dyes and radioactive colloids, thereby reducing detection rates and increasing false negative rates for SLNBs [22, 26]. The risks associated with ALND and TAD include axillary pain, nerve damage, lymphoedema, and sensory dysfunction, and patients with pLCR will not benefit from undergoing these procedures [27, 28]. Therefore, accurately identifying ALN status after NAC in patients with BC is crucial for improving prognosis predictions and treatment planning.

Currently, radiomic methods for tumour diagnosis, treatment guidance, and prognosis prediction are developing rapidly [29]. MRI provides a complete picture of the entire breast tumour, and MRI-based radiomics is a key area of exploration [30]. Some researchers have already proposed combining breast MRI characteristics with tumour stage, HR status, and HER2 status to predict whether pLCR will be achieved [31]. Super-resolution reconstruction technique can break through the physical limitations of MRI images by technical means, which can highlight tiny lesions (e.g., early tumours, microcalcified foci) and help imaging histology models capture finer biological features, while improving the robustness and reproducibility of the features, and enhancing the sensitivity of imaging histology in the prediction of therapeutic efficacy [32, 33]. However, breast tumours are heterogeneous in time, space, and molecular subtypes. We chose to explore the efficacy of NAC in treating metastatic ALNs in patients with luminal BC. In this study, we constructed models based on MRI radiological features and clinicopathological characteristics using eight machine-learning algorithms, and the resulting multi-omic prediction model accurately and effectively predicted ALN status after NAC in the patients with luminal BC.

The TIME in BCs is a complex system comprising multiple cell types and molecules, and the interactions between these factors affect treatment efficacy. Due to the differences in the quantity and quality of the immune population, patients who experience differing treatment efficacies have unique immune landscape characteristics [34]. In recent years, treatment strategies targeting the TIME have become a hot research topic in BC, aiming to improve treatment efficacy by identifying targetable TIME differences [35, 36]. Our findings suggest that the group with poor NAC outcomes had immunosuppressive characteristics, including greater proportions of unactivated naive T cells (such as CD4 naive T cells) and fewer cytotoxic T cells (such as CD8 Trm cells) in the BC TIME. We also found higher proportions of immunosuppressive macrophage subclusters, such as macrophages_LYVE1 and macrophage_CXCL1, and the immunosuppressive fibroblast_2 subcluster in the nLCR group (Supplementary Results). These results suggest an immunosuppressive mechanism underlying ALN resistance to NAC and present a direction for future research.

Furthermore, our analysis identified some notable potential target genes. For example, the CD4Tfh_CXCL13 subcluster formed a considerable fraction in the nLCR group, whereas the CD4Treg_FOXP3 subcluster was predominant in the ITC group. Compared with other subclusters, these two expressed high levels of TIGIT, a common immunosuppressive molecule. TIGIT acts as a co-inhibitory receptor that indirectly inhibits immune cells and suppresses the immune response against tumours. In particular, TIGIT can bind CD155 on dendritic cells, which reduces IL-12 secretion and increases IL-10 release, thereby indirectly hindering T-cell activity [37].

Another potential target pathway was identified in our findings regarding the CD8Trm_ZNF683 subcluster, which was more commonly found in the pLCR subgroup than in the other subgroups. This cell type releases high levels of multiple chemokines, which leads to recruitment of immune cells to the tumour site. This cell type also expresses high levels of the *CRTAM* gene, which can interact with CADM1 to promote NK cell cytotoxicity and IFN-γ secretion by CD8+ T cells in vitro. NK cell-mediated rejection of CADM1-expressing tumours is also influenced by the *CRTAM* gene in vivo, thus regulating the activation of various T-cell subtypes [38].

Nowadays, there is no effective way to preoperatively assess the neoadjuvant chemotherapy response of metastatic axillary lymph node. While some journals have published studies on radiomics, machine learning applications in breast cancer neoadjuvant chemotherapy response prediction, the integration of these methodologies with single-cell analyses to specifically predict neoadjuvant chemotherapy response of metastatic axillary lymph node and analyze tumor immune microenvironment changes in luminal breast cancer is a novel contribution.

However, there are some limitations. First, the sample size of scRNA-seq analysis was small and the depth of cell clustering analysis was insufficient, Several identified clusters represent atypical populations or are defined by only a single marker gene. Secondly, although we validated the results of single-cell data analysis by multiplex immunofluorescence at the protein level in patient specimens, these interpretations are still largely speculative, which lacked the support of sufficient molecular and functional characterization, we will further validate them in the direction of mechanism by basic experiments. Finally, this paper is a small-sample retrospective study, and we will conduct prospective clinical validation in the future, hoping to further enhance the scientific rigor and clinical relevance of this study.

## Conclusion

In this study, we innovatively used SR-MRI images and clinical data to establish a multi-omics prediction model to assess the effectiveness of NAC in patients with luminal BC and ALN metastases; the model demonstrated good overall performance. Subsequently, we performed scRNA-seq analysis on a subset of patients with disparate treatment responses to explore the association between TIME and NAC efficacy. This provided a theoretical basis for a potential mechanism underlying resistance against NAC in treating ALN metastases. This theoretical mechanism underlying resistance presents a possible avenue for exploration to provide improved personalised treatment guidance.

## Methods

### Study design and patient selection

In this study, we selected patients with BC who received NAC between February 2022 and August 2024 from two separate centres, the Affiliated Hospital of Qingdao University for the training cohort and the Affiliated Hospital of Jining Medical College for the validation cohort. Clinical, pathological, and imaging data were retrospectively collected. The inclusion criteria were: (1) female patients who were ≥18 years old; (2) invasive luminal BC that was histologically confirmed prior to any treatment; (3) metastasis in the ipsilateral ALNs confirmed by preoperative puncture biopsy; (4) complete breast DCE-MRI pre- and post-NAC; and (5) post-NAC ALND with clear postoperative pathology. The exclusion criteria were: (1) a history of surgery or radiation therapy in the ipsilateral axilla, including SLNB performed prior to NAC initiation; (2) distant organ metastasis; (3) previous or concurrent malignant tumours; (4) failure to complete the full cycle NAC regimen; (5) endocrine therapy or immunotherapy prior to surgery; and (6) bilateral BC.

Ultimately, this study comprised 457 patients, of which 375 were part of the training cohort and 82 were part of the validation cohort. From the training cohort, 14 pre-treatment samples (including five primary tumours, four paracancerous tissues, and five metastatic ALNs) from five patients were obtained for scRNA-seq. The study flowchart is shown in Fig. 1A, B. The Affiliated Hospital of Qingdao University Ethics Committee approved this study (approval number QYFY-WZLL-27822), and tissue samples were obtained with written informed consent from each patient.

### Treatment, pathological confirmation, and outcome measures

All patients received six or eight cycles of anthracycline- and taxane-based drugs before surgery. NAC was followed by breast-conserving surgery or mastectomy and ALND. Pathological type was diagnosed based on immunohistochemical analysis of preoperative puncture biopsy samples, with luminal BC defined as HR-positive (oestrogen receptor or progesterone receptor nuclear staining ≥1%) and HER2 negative (fluorescence in situ hybridisation negative) [23]; Ki-67 was divided into high- and low-expression groups using a cut-off point of 20% expression. Pathological outcomes were based on postoperative pathology and included pBCR, defined as no invasive cancer in the primary breast lesion (ypT0/is); pLCR, defined as no malignant tumour cells in the ALNs (ypN0); and pCR, defined as both pBCR and pLCR (ypT0/isN0M0). Radiological outcomes were based on post-NAC MRI and included rBCR (no residual lesions in the breast) and rLCR (no evidence of residual disease in the axilla). We collected clinical data, including age at diagnosis, menopausal status, pathology type, clinical T and N stages, histologic grading, Ki67 expression, TILs, rBCR, and rLCR.

### MRI acquisition and feature selection

#### Imaging and processing

MRI was completed before NAC initiation and within 2 weeks after termination (Supplementary Table 1), and DCE sequences were primarily used here. For contrast-enhanced MRI, intravenous Gd-DTPA (0.2 mmol/kg, flow rate 2.0 mL/s) was used and enhanced scanning was performed 90, 180, 270, and 360 s after injection. The acquired DCE-MRI breast images were resampled for uniform voxel spacing (1 × 1 × 1 mm^3^); N4 bias field correction was performed using the Python package SimpleITK (v2.0.2); and *Z*-score normalisation was applied. SR reconstruction was performed using a deep learning generative adversarial network to enhance image resolution. SR images were obtained using migration learning with the Onekey platform (v 3.1, Beijing, China), which can increase the resolution by a factor of four while maintaining the size of the original image (Supplementary Methods 1).

#### Tumour segmentation and radiomics feature selection

ITK-SNAP (v4.0.2) was used to manually segment the processed images to delineate the primary tumour area layer by layer while avoiding necrotic and liquefied areas of the tumour [39]. Two radiologists with over 5 years of expertise in diagnostic breast MRI performed the segmentation. A third radiologist with 10 years of experience in diagnostic breast imaging settled any disagreement. The open-source Python package Pyradiomics (v3.0) was used to extract RFs of the outlined tumour [40]. The extracted RFs included geometric, intensity, and texture features. Next, we filtered the dimensions of the extracted RFs. Further details regarding RFs and filtering methods are described in Supplementary Methods 2 and 3.

### Prediction model establishment

The construction and evaluation of single-modality radiomics model was performed based on the Python package scikit-learn (v0.20). The training cohort was used for model construction. We trained a total of eight machine-learning models: logistic regression, SVM, k-nearest neighbours, random forest, extremely randomised trees, extreme gradient boosting, light gradient boosting machine, and multi-layer perception, using the bootstrap method. To select the optimal model hyperparameters for fitting the models, the models were developed using a sub-training cohort (80%) and hyperparameter tuning was evaluated using a sub-validation dataset (20%) in each repeated training iteration. To prevent overfitting of the model, we used L2 regularisation and 10-fold cross-validation framework. To ensure model robustness, we repeated the entire build process 1000 times using the bootstrap method. An optimal model was finally determined to be validated using the validation cohort. Finally, we used the DeLong test to compare the AUC, specificity, sensitivity, accuracy, and positive and negative predictive values of each model, and calculated the radiomic score (rad-score) (Supplementary Methods 4).

Clinicopathological characteristics were screened for independent predicting factors using univariate and multivariate logistic regression analysis. Unimodal radiomic, unimodal clinical, and multi-omic prediction models were constructed. The accuracy of the nomogram was assessed using a calibration curve, and the goodness-of-fit was assessed using the Hosmer–Lemeshow test. The nomogram’s discriminatory ability was assessed using the AUC of receiver operating characteristic metric. Decision curve analysis was conducted by figuring out the nomogram’s net benefits.

### Single-cell RNA library preparation, sequencing, and data analysis

To explore TIME heterogeneity in patients with differing ALN responses to NAC, we performed scRNA-seq on a total of 14 samples of tumour tissue (T), paired paracancerous tissue (P), and ALNs (L) from five pre-NAC patients. These were categorised into nLCR and pLCR groups based on the postoperative pathology results. Of these, one patient showed only isolated tumour cells in the ALNs, and we placed this patient into a separate group (ITC). The nLCR group comprised a total of six samples from patients 1 and 2, including nLCR_T (T1, T2), nLCR_P (P1, P2), and nLCR_L (L1, L2); the ITC group comprised two samples from patient 3, including ITC_T (T3) and ITC_L (L3); the pLCR group comprised six samples from patients 3 and 4, including pLCR_T (T4, T5), pLCR_P (P4, P5), and pLCR_L (L4, L5) (Fig. 1C). Preparation and sequencing of single-cell RNA library, quality control, dimension-reduction, clustering, and then correlation analysis was conducted. Detailed description of analysis is shown in Supplementary Methods 5.

### Multiplex immunofluorescence (mIF) staining and analysis

Formalin-fixed paraffin-embedded (FFPE) sections (3 μm) were used for the analysis. Staining was performed using the AlphaTSA Multiplex Kit. Briefly, following deparaffinization and rehydration, sections underwent antigen retrieval in a retrieval buffer (pH 9.0) at 95 °C for 20 min. Subsequently, sections were treated for endogenous peroxidase blocking, incubated with a blocking buffer, and then subjected to sequential antibody incubation. This process involved primary antibody incubation, binding of a horseradish peroxidase (HRP)-conjugated secondary antibody, and signal amplification with an XTSA dye. The antibody panel included CD3 (1:100 dilution, ab16669, abcam), CD4 (1:1600 dilution, R50028, zenbio), FOXP3 (1:3000 dilution, ab20034, abcam), TIGIT (1:100 dilution, TA812978, Thermo Fisher Scientific), CD68 (1:200 dilution, GM087629, ecotek), LYVE1 (1:5000 dilution, ab219556, abcam), and CD163 (1:400 dilution, ab182422, abcam).After staining for all targets was complete, DAPI was used for nuclear counterstaining. Whole-slide images were acquired using a PathScan slide scanner. The image analysis workflow consisted of spectral unmixing using InForm® software, followed by importing the images into HALO® software (Indica Labs) for quantitative cell analysis.

### Statistical analysis

The Student’s *t*-test and the chi-squared test were used to compare continuous and categorical data, respectively. Continuous data are given as mean and standard deviation; categorical variables are provided as numbers and percentages. For both univariate and multivariate trials, logistic regression analysis was used to calculate hazard ratio (HRs) with 95% confidence intervals. *P* < 0.05 was deemed statistically significant. Python (v3.9) and R statistical software (v 4.3.1) were used for statistical analysis and visualisation.

## Supplementary information

Supplementary information

## Data Availability

The datasets analysed during the current study available from the corresponding author on reasonable request.
