# Supplementary material for: Multi-omics prediction of axillary treatment response and tumour microenvironment alterations in lymph node-positive luminal breast cancer
Source: Cell Death Dis. 2025 Aug 4;16(1):588. doi: 10.1038/s41419-025-07877-6 (PMC12322124; doi:10.1038/s41419-025-07877-6)
Supplement: Supplementary file 1 — Supplementary information [file 41419_2025_7877_MOESM1_ESM.doc]

**Supplementary Method**

**Supplementary Method 1: Super-resolution reconstruction：**

Denoising the raw MRI images and removing any artefacts was the first step in the preparation phase of Onekey's super-resolution reconstruction technique. Onekey used Non-Local Means Denoising as a noise reduction technique. By analysing the similarities between the tiny regions around each pixel and other portions of the image, this technique lowers noise while maintaining the image's features and structure. In terms of parameter configurations, the similarity window is 11 × 11 pixels in size, and the search window is 21 × 21 pixels. In order to maximise the denoising impact and avoid too smoothing off visual features, these values have been carefully chosen. An adaptive threshold approach, which detects brightness irregularities in specific regions of the image brought on by a variety of causes, is used to remove artefacts. For local smoothing of these detected aberrant regions, Onekey uses a Gaussian kernel standard deviation of 1.5 and applies a small amount of Gaussian blurring. This stage lessens the effect of artefacts on the final image quality.

Next, Onekey made ensured that the strength values were all on the same scale by normalizing them across all the pictures. This is important for making sure that everything is the same in later processing steps. Onekey used the Min-Max Normalization method to make the sharpness values in our pictures more consistent. This method changes the range of the original image data to a standard scale of [0, 1]. This normalization method essentially makes the pixel intensities the same in all pictures, getting rid of differences that could be caused by different scanning devices or settings. This makes sure that the picture data is consistent and can be compared during the super-resolution rebuilding process.

Onekey also applied Gaussian white noise and Gaussian fuzz to the images, lowering the resolution to a quarter of the original along the X and Y axes on purpose. This made low-resolution MRI pictures that could be used in the super-resolution process.

In super-resolution restoration, OneKey merged a generative adversarial network (GAN) with a deep transfer learning network. It is the goal to improve spatial resolution by turning low-resolution MRI pictures into high-resolution ones.

The core architecture of the 3D SR reconstruction technique is a Generative Adversarial Network (GAN). A discriminator network and a generator network make up a GAN, a deep learning model. Low-resolution pictures are fed into the generator, which uses many convolutional layers (Conv2D) with batch normalization (BatchNorm) and ReLU activation functions to extract features from the images. The Tanh activation function is utilized in the generator's last layer to produce high-resolution pictures. High-resolution pictures are sent into the discriminator, which employs numerous convolutional layers (Conv2D) with Dropout layers and LeakyReLU activation functions to assess the images' validity. Lastly, the discriminator outputs a binary classification (natural or created) using a Sigmoid activation function. The training process for these two networks is adversarial. While the discriminator tries to correctly discern between created and real pictures, the generator tries to produce images that can fool the discriminator. The generator network can successfully learn the association between low-resolution and high-resolution pictures because to this adversarial training procedure. A GAN model is the end product of this training.

The spatial resolution of low-resolution MRI images may be greatly improved by converting them into high-quality pictures using the trained GAN model. The original MRI picture is first segmented into 128 × 128 input patches using Onekey. Second, each 128 × 128 pixel low-resolution patch is sent into the generator, which creates a 512 × 512 pixel high-resolution picture in return. To recreate the entire high-resolution MRI picture, Onekey then combined all of the produced high-resolution patches in accordance with their initial locations.

By using this method, the voxel size was adjusted, going from its initial 1 × 1 × 1mm^3^ dimensions to a more precise resolution of 0.25 × 0.25 × 1mm^3^. As seen in Supplementary Figure 1, this enhancing step produced super-resolution MRI images using SR reconstruction.

**Supplementary Method 2: Details of radiomics features**

Geometric features describe the three-dimensional shape of the tumor. Intensity features describe the first-order statistical distribution of voxel intensities within the tumor. Texture features describe the pattern and spatial distribution of intensities, including second-order and higher-order patterns. Texture features include the gray-level co-occurrence matrix (GLCM) features, gray Level Dependence Matrix (GLDM) features, gray-level run length matrix (GLRLM) features, gray-level size zone matrix (GLSZM) features and neighborhood gray-tone difference matrix (NGTDM) features.

**Supplementary Method 3: Methods of filtering RFs**

First, all RFs were statistically tested using the Mann–Whitney U test, and only features with a correlation of P<0.05 were retained. Second, we calculated the correlation between characteristics using the Spearman rank correlation coefficient to identify features with good repeatability. To preserve the descriptive power of the RFs to the greatest extent, RFs were retained if the correlation between any two features was >0.9; with each iteration, the RF with the greatest redundancy was deleted. Lastly, the least absolute shrinkage and selection operator (LASSO) regression algorithm was performed under R package glmnet to select relevant RFs with non-zero coefficients from the training cohort; We used 10-fold cross-validation to adjust the optimal λ value, choosing the λ that lead to the smallest cross-validation error.

**Supplementary Method 4: Rad-score calculation method：**

Rad-score =

0.2664835164835164 - 0.059157×original_shape_Sphericity

+ 0.029868×original_shape_SurfaceVolumeRatio

- 0.013422×wavelet_HHH_firstorder_Kurtosis

+ 0.025750×wavelet_HHL_firstorder_Mean

+ 0.000633×wavelet_HLH_glcm_ClusterProminence

+ 0.006767×wavelet_HLH_glszm_SmallAreaEmphasis

+ 0.013766×wavelet_HLL_glcm_ClusterShade

+ 0.016563×wavelet_HLL_glszm_SmallAreaEmphasis

+ 0.023180×wavelet_LHH_glszm_SmallAreaEmphasis

- 0.026524×wavelet_LHL_glcm_Correlation

- 0.009353×wavelet_LLH_ngtdm_Contrast

+ 0.016428×wavelet_LLL_firstorder_Minimum

**Supplementary Method 5: Single-cell RNA library preparation, sequencing, and data analysis**

5.1 | Single-cell RNA library preparation and sequencing

A Singleron PythoN® Automated Tissue Dissociator (Singleron Biotechnologies, Nanjing, China) and sCelLive® Tissue Dissociation Mix (Singleron Biotechnologies) were used to separate tissues from puncture biopsies into single-cell suspensions. Cell viability was evaluated under a microscope using trypan blue staining.

Microfluidic devices were loaded with single-cell suspensions at a density of 2 × 10^5^ cells/mL of PBS. Following the Singleron GEXSCOPE® methodology, scRNA-seq libraries were created using the Singleron Matrix® Automated single-cell processing equipment and the GEXSCOPE® Single-Cell RNA Library Kit (Singleron Biotechnologies). Libraries were pooled for sequencing after being diluted to 4 ng/µL. Using 150 bp paired-end reads, pools were sequenced with the Illumina Novaseq 6,000 apparatus (Illumina, Inc., San Diego, CA, USA).

5.2 | Primary analysis of raw read data

CeleScope (v1.5.2) (Singleron Biotechnologies, Nanjing, China) was used to build gene expression profiles from the raw read data using standard parameters. From R1 readings, barcodes and unique molecular identifiers (UMIs) were taken out and modified. R2 reads were edited to remove adapter sequences and poly A tails before being matched against the GRCh38 (hg38) transcriptome using STAR (v2.6.1b). FeatureCounts (v2.0.1) was subsequently used to allocate the uniquely mapped readings to exons. The same gene, UMI, and successfully assigned readings were merged to form a gene expression matrix for further investigation.

5.3 | Quality control, dimension-reduction, clustering and plotting

Python package Scanpy (v1.9.3) was utilised for quality control, dimensionality reduction, clustering, and plotting. We used the following exclusion criteria to filter the expression matrix for every example dataset: (1) cells with <200 genes or those in the top 2% of genes; (2) cells with a UMI count in the top 2%; (3) genes expressed in <5 cells; and (4) cells with a mitochondrial concentration <20%. A total of 86,279 cells with an average of 1,002 genes and 2,611 UMIs per cell were retained after filtering for further study.

The raw count matrix was logarithmically converted into a normalised data matrix, with the total counts in each cell being used for normalisation.

The R package Seurat (v4.1.0) was utilised to select the top 2,000 variable genes. The scaled variable gene matrix was subjected to principal component analysis. The top 20 principal components were then used for dimensional reduction and grouping. With a resolution value of 0.8, the cells were divided into 11 clusters using the Louvain method; these were visualised using the UMAP approach.

5.4 | DEG analysis

The Seurat FindMarkers function, which is based on the Wilcoxon rank sum test with default settings, was used to select DEGs. Genes with an average log (fold change) value greater than 0.25 and expression in more than 10% of cells in both groups were classified as DEGs. A 0.05 standard cutoff was applied to assess the statistical significance of the adjusted P value, which was computed using the Bonferroni correction. Based on the conventional cell marker patterns of expression, cell doublets were calculated. For further examination, any clusters enriched with more than one marker unique to a certain cell type were disregarded.

5.5 | Pathway enrichment analysis

To provide insight into the possible roles of DEGs, Gene Ontology (GO) data were analysed utilising the R package ClusterProfiler (v4.0.0). Pathways with P_adj <0.05 were deemed to be substantially enriched, and bar plots were used to display major pathways. To find pathway enrichment, the gene set variation analysis was performed using the average gene expression of each cell type as input data. GO gene sets encompassing the cellular component, biological process, and molecular function categories were utilised as a point of reference.

5.6 | Cell type annotation and subtyping of major cell types

Each cluster’s cell types were identified by searching the reference database SynEcoSys® (Singleron Biotechnology) for canonical markers. For single-cell sequencing data, SynEcoSys® provides collections of canonical cell type markers from CellMakerDB, PanglaoDB, and newly published literature.

To create a high-resolution map of every cluster, cells were taken from each cluster and reanalysed using the same techniques for a more in-depth examination, setting the clustering resolution at 0.8.

5.7 | Functional gene module analysis

Hotspot analysis was used to identify functional gene modules that exhibit variability among subpopulations of epithelial cells. Briefly, the “danb” model was used to identify the modules of the top 500 genes with the greatest autocorrelation Z-score . The create_modules function was then used to identify the modules, with min_gene_threshold=15 and fdr_threshold=0.05. The calculate_module_score function was used to determine the module score.

5.8 | UCell gene set scoring and metabolic activity analysis

The R package UCell (v2.2.0) was utilised to score the gene sets. By classifying query genes according to expression levels in distinct cells, the Mann–Whitney U test was used to calculate UCell scores. R package ScMetabolism (v0.2.1) was used to quantitatively assess single-cell metabolic activity. ScMetabolism collates Kyoto Encyclopedia of Genes and Genomes metabolic pathways and calculates metabolic pathway enrichment scores using VISION arithmetic. Scores for specific pathways were visualised using the FeaturePlot and VlnPlot functions in Seurat and pheatmap.

5.9 | Developmental trajectory inference

R package Monocle2 (v2.22.0) was used to recreate the cell differentiation trajectory. Highly variable genes were identified with the Seurat FindVariableFeatures, and DDRTree performed dimension reduction. The Monocle2 plot_cell_trajectory function displayed the trajectory. Furthermore, the Python package CytoTRACE (v0.3.3) was used to predicted the differentiation potential of monocyte subpopulations, a computational method based on gene counts and expression data from scRNA-seq that predicts stages of cell differentiation.

**Supplementary Results**

Heterogeneity of fibroblasts

In this analysis, fibroblasts were categorised into a total of six subclusters (Supplementary Fig. 6A–C). Of these, the fibroblast_1 subcluster was primarily from the pLCR group. Compared with other subclusters, fibroblast_1 has a higher score for extracellular matrix remodelling and myofibroblast characteristics and highly expressed the *ADH1B* gene (Supplementary Fig. 6D–F). The fibroblast_2 subcluster was predominantly derived from the nLCR group and highly expressed the *IGFBP2* gene (Supplementary Fig. 6F). The fibroblast_5 subcluster was mainly derived from the ITC samples. Compared with other subclusters, fibroblast_5 had higher scores for extracellular matrix remodelling and myofibroblast characteristics. It highly expressed a variety of matrix metalloproteinase (MMP)-related genes, such as *MMP11* and *MMP14* (Supplementary Fig. 6D–F). In summary, varying degrees of immunosuppression in fibroblasts may have led to differences in NAC efficacy in ALN metastases.

**Supplementary Tables**

**Supplementary Table1. Detailed information and parameters of the MRI scanner**

| Cohorts | Train | Validation |
| --- | --- | --- |
| Scanner | GE | GE |
| Magnetic field strength | 3.0T | 3.0 T |
| Sequence name | DCE | DCE |
| TR/TE(ms) | 4.6/2.1 | 7.2/1.2 |
| Flip angle (°) | 10 | 110 |
| Field of view (mm × mm) | 380×380 | 340×340 |
| Scan matrix | 320 × 320 | 320 × 320 |
| Slice thickness (mm) | 1 | 0.7 |
| Number of slices | 140 | 30 |
| Slice Gap (mm) | 0.5 | 0 |

**Supplementary Table2. Performance of the radiomics models for predicting pLCR in the training and validation cohorts**

|  | Accuracy | AUC | 95% CI | Sensitivity | Specificity | PPV | NPV |
| --- | --- | --- | --- | --- | --- | --- | --- |
| LR, training | 0.69 | 0.76 | 0.69–0.82 | 0.74 | 0.67 | 0.45 | 0.88 |
| LR, validation | 0.73 | 0.76 | 0.65–0.87 | 0.75 | 0.72 | 0.50 | 0.88 |
| SVM, training | 0.81 | 0.86 | 0.81–0.92 | 0.88 | 0.79 | 0.60 | 0.95 |
| SVM, validation | 0.58 | 0.71 | 0.58–0.84 | 0.85 | 0.47 | 0.38 | 0.89 |
| KNN, training | 0.80 | 0.85 | 0.81–0.90 | 0.56 | 0.89 | 0.65 | 0.85 |
| KNN, validation | 0.66 | 0.66 | 0.54–0.79 | 0.55 | 0.70 | 0.41 | 0.80 |
| RandomForest, training | 0.76 | 0.86 | 0.82–0.91 | 0.84 | 0.72 | 0.52 | 0.93 |
| RandomForest, validation | 0.71 | 0.77 | 0.66–0.88 | 0.70 | 0.72 | 0.48 | 0.86 |
| ExtraTrees, training | 0.64 | 0.76 | 0.70–0.82 | 0.82 | 0.58 | 0.41 | 0.90 |
| ExtraTrees, validation | 0.75 | 0.75 | 0.63–0.88 | 0.55 | 0.83 | 0.55 | 0.83 |
| XGBoost, training | 0.80 | 0.94 | 0.91–0.97 | 0.95 | 0.75 | 0.58 | 0.98 |
| XGBoost, validation | 0.75 | 0.73 | 0.59–0.86 | 0.60 | 0.81 | 0.55 | 0.84 |
| LightGBM, training | 0.77 | 0.87 | 0.82–0.91 | 0.83 | 0.74 | 0.54 | 0.92 |
| LightGBM, validation | 0.77 | 0.73 | 0.61–0.86 | 0.50 | 0.87 | 0.59 | 0.82 |
| MLP, training | 0.68 | 0.77 | 0.71–0.83 | 0.82 | 0.63 | 0.44 | 0.91 |
| MLP, validation | 0.75 | 0.78 | 0.66–0.89 | 0.70 | 0.77 | 0.54 | 0.87 |

The eight machine learning algorithms were as follows: logistic regression (LR), support vector machine (SVM), k-nearest neighbors (KNN), random forest (RF), extremely randomized trees (ExtraTrees), extreme gradient boosting (XGBoost), light gradient boosting machine (LightGBM), and multi-layer perception (MLP).

AUC, area under the curve; CI, confidence interval; NPV, negative predictive value; pLCR, pathological lymph node complete response; PPV, positive predictive value.

**Supplementary Table3. Univariate and multivariate analyses of clinical and pathological factors**

|  | Univariate logistic regression | | Multivariate logistic regression | |
| --- | --- | --- | --- | --- |
|  | OR (95% CI) | *P* value | OR (95% CI) | *P* value |
| Age 35 years | 1.633 (0.539–4.950) | 0.386 | NA | NA |
| Menopause status | 0.762 (0.480–1.209) | 0.249 | NA | NA |
| Pathology | 1.840 (0.979–3.455) | 0.058 | NA | NA |
| T stage | 0.707 (0.505–0.991) | 0.044 | 0.760 (0.523–1.105) | 0.151 |
| N stage | 0.211 (0.122–0.366) | 0.000 | 0.213 (0.120–0.378) | 0.000 |
| Grade | 0.547 (0.301–0.995) | 0.048 | 0.529 (0.274–1.021) | 0.058 |
| Ki-67 | 0.741 (0.466–1.180) | 0.207 | NA | NA |
| TILs | 2.354 (1.406–3.940) | 0.001 | 2.252 (1.271–3.989) | 0.005 |
| yBCR | 3.726 (1.555–8.925) | 0.003 | 2.687 (1.030–7.012) | 0.043 |
| yLCR | 3.295 (1.687–6.436) | 0.000 | 3.047 (1.434–6.475) | 0.004 |

CI, confidence interval; Ki-67, proliferation marker protein Ki-67; OR, odds ratio; TILs, tumor infiltrating lymphocytes; yBCR, post-neoadjuvant therapy breast complete response; yLCR, post-neoadjuvant therapy lymph node complete response.

**Supplementary Figures**

**Supplementary Figure 1. Original image and Super-resolution reconstruction image**
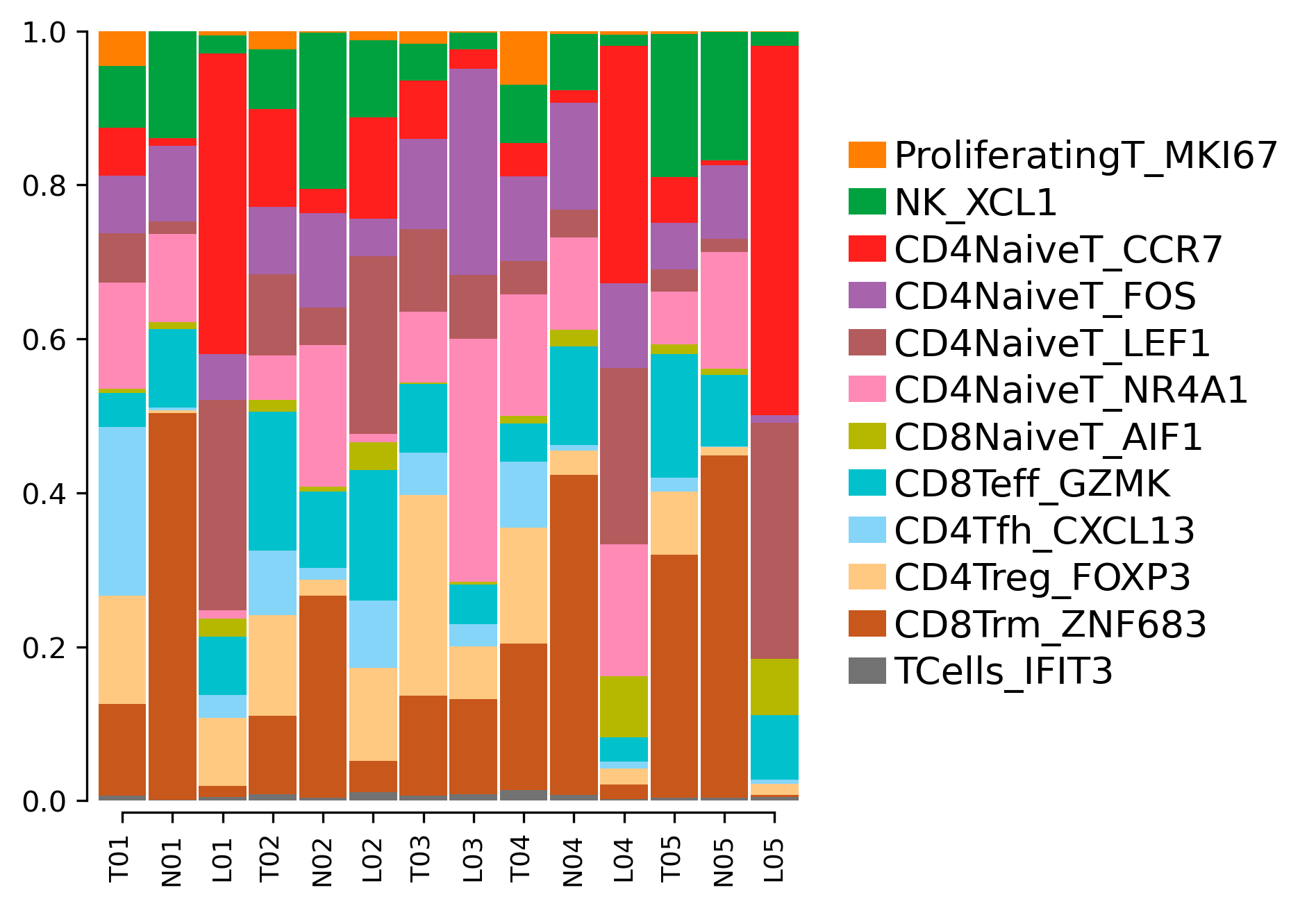


(A). Distribution of T and NK cells in all samples.


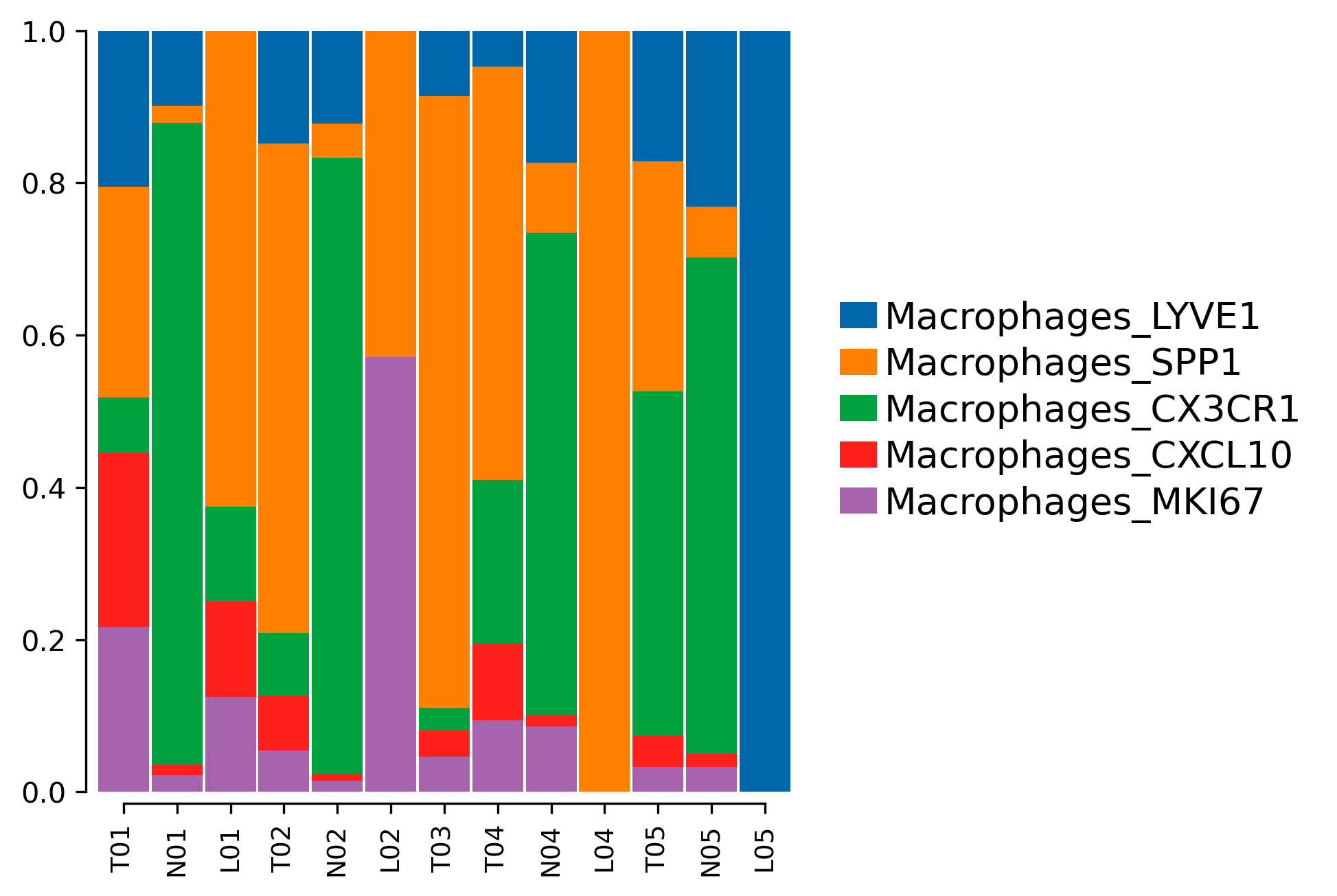


(B).Distribution of mononuclear phagocytes in all samples.


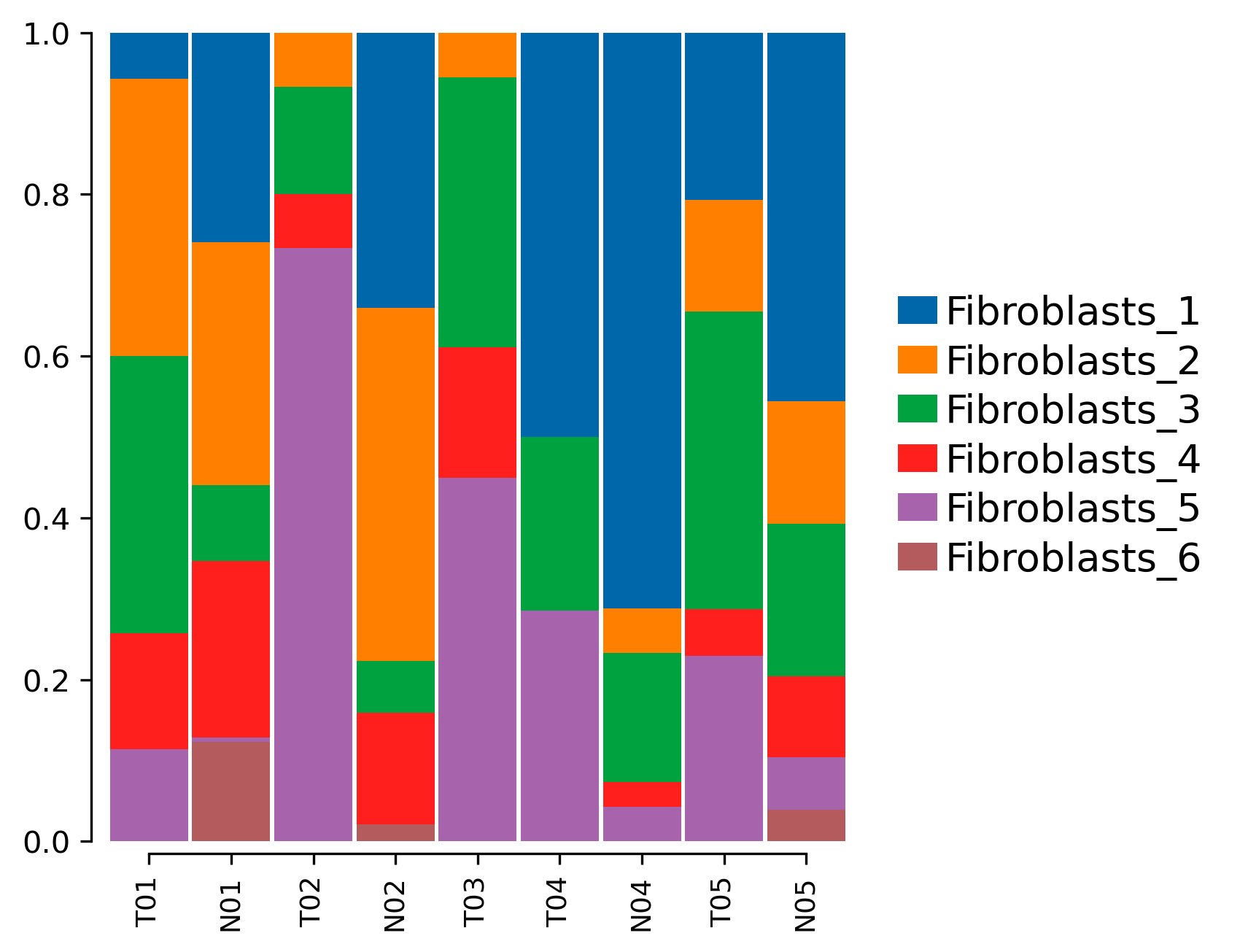


(C).Distribution of fibroblasts in all samples.

**Supplementary Figure 2. Original image and Super-resolution reconstruction image**


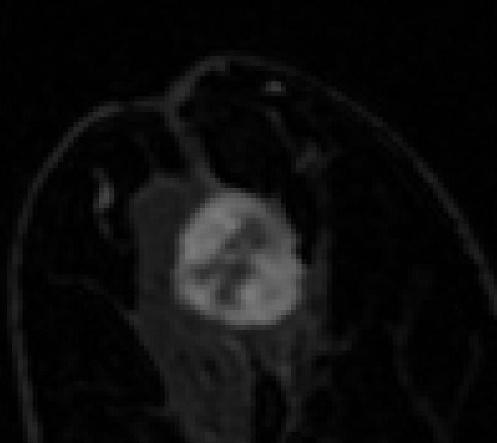

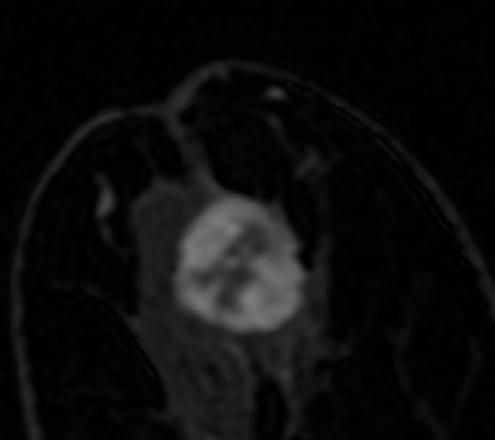


(A). Original image (B).Super-resolution reconstruction image

**Supplementary Figure 3. Details of all radiomic features**


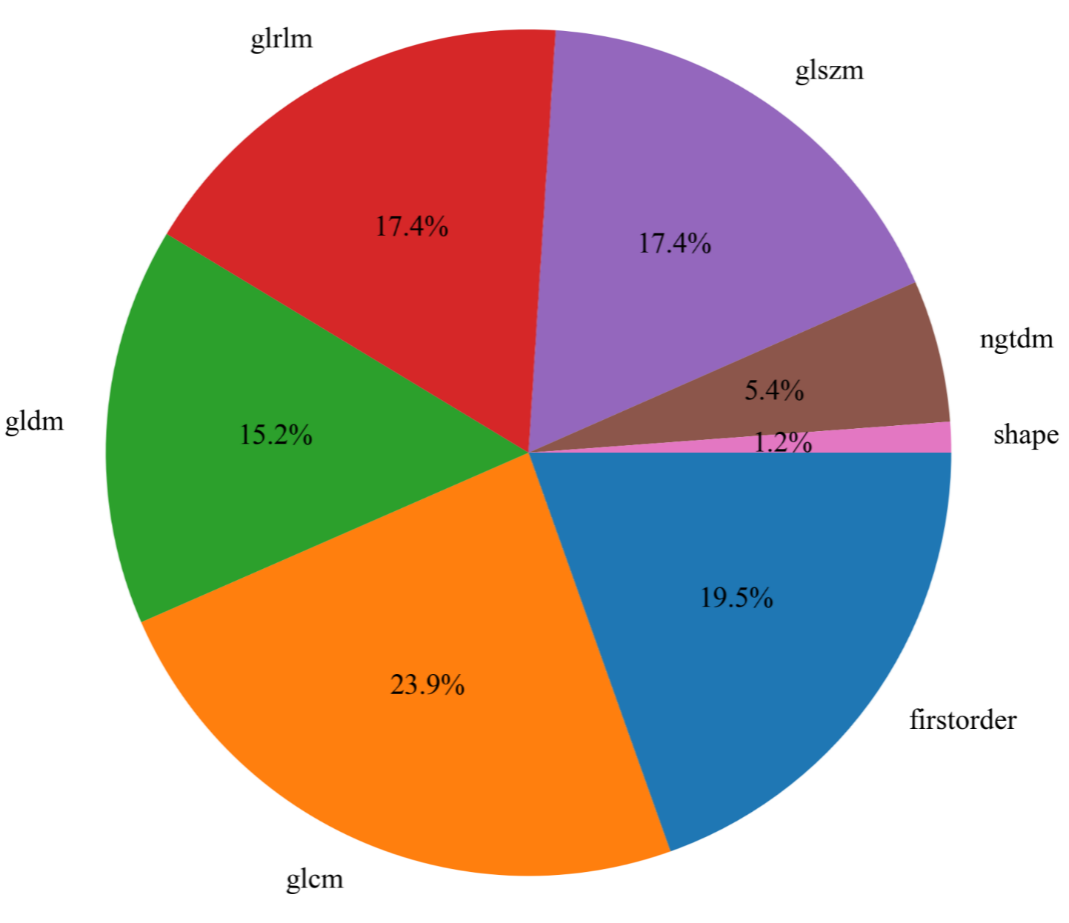


(A) Distributions of radiomic features


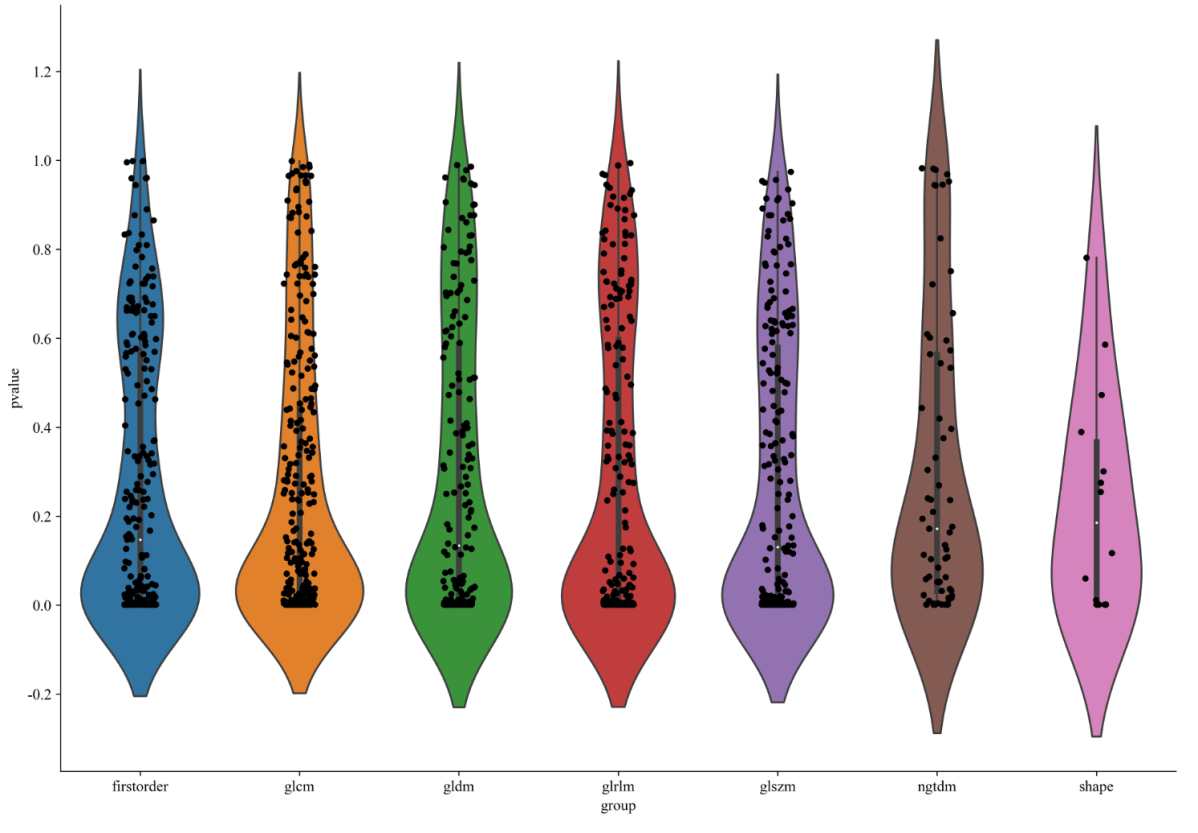


1. Distributions of radiomics feature correlation.

**Supplementary Figure 4. Details of selected radiomic features**


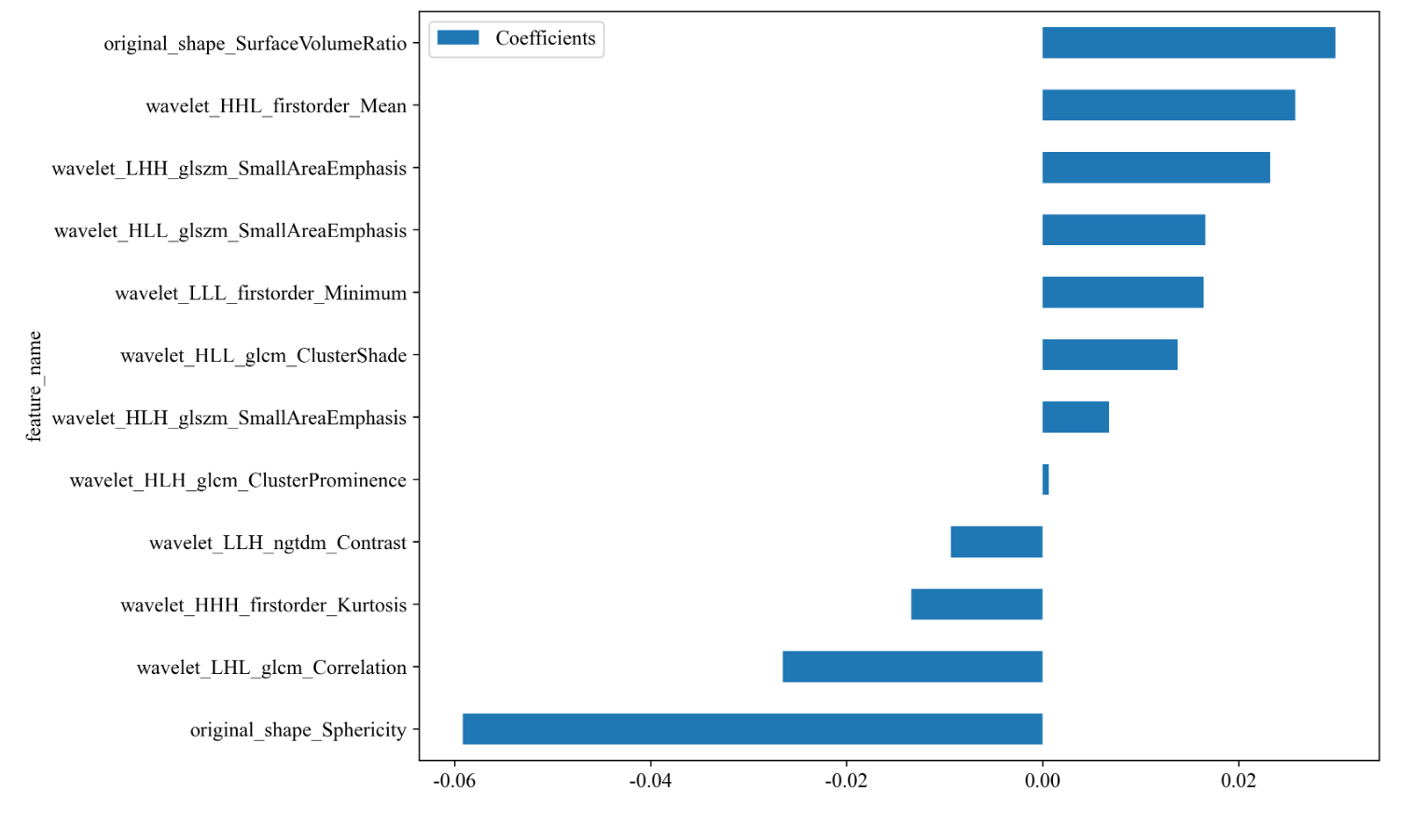


**Supplementary Figure 5. Validation of the multimodal prediction model.**

**
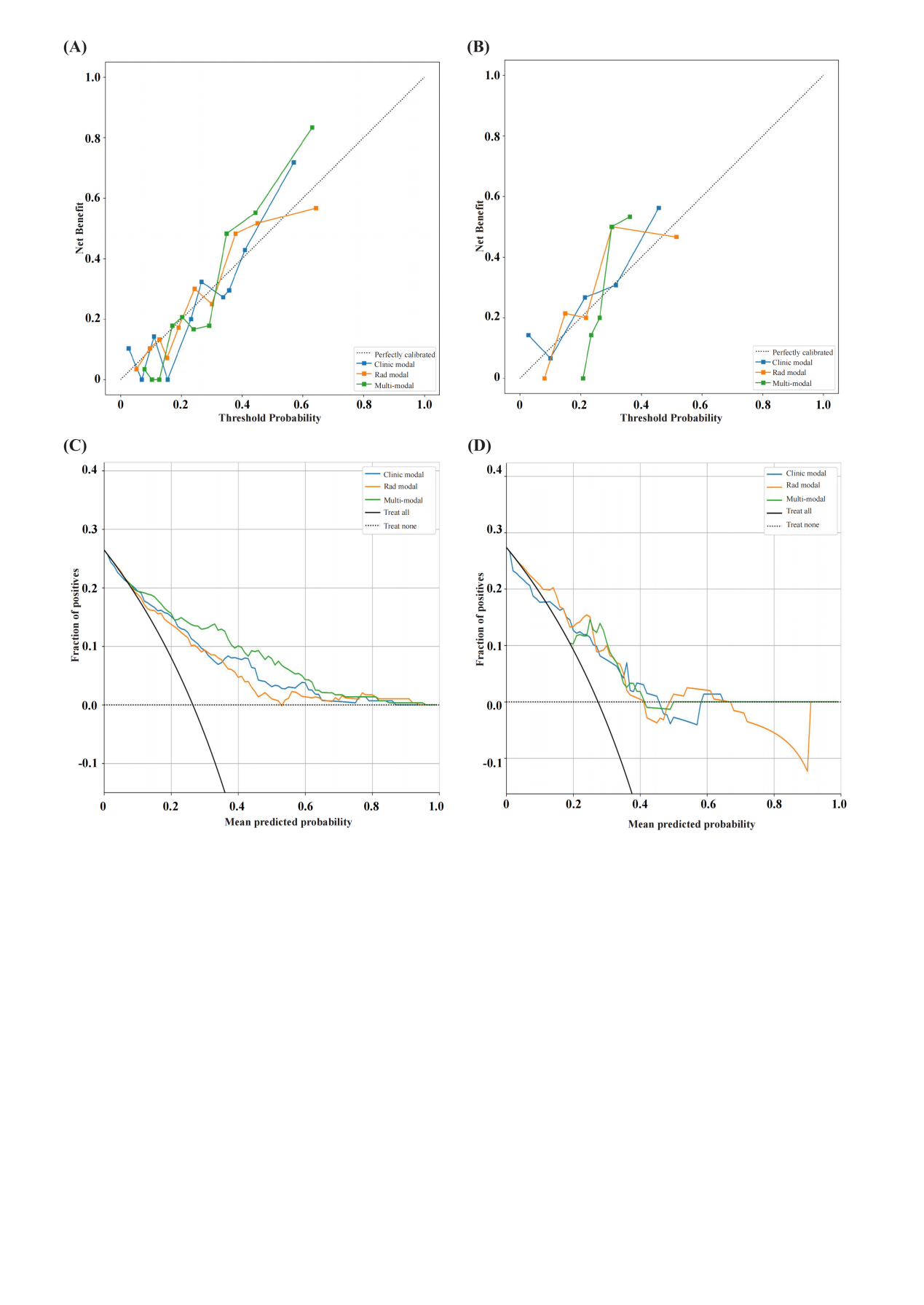
**

(A) The calibration curves to predict pLCR in the training cohort. (B) The calibration curves to predict pLCR in the validation cohort. (C) DCA curves of the nomogram in the training cohort. (D) DCA curves of the nomogram in the validation cohort. DCA, decision curve analysis; pLCR, lymph node pathological complete response.

**Supplementary Figure 6. Reclustering of fibroblasts isolated from 14 samples from 5 patients with differing axillary responses after NAC.**


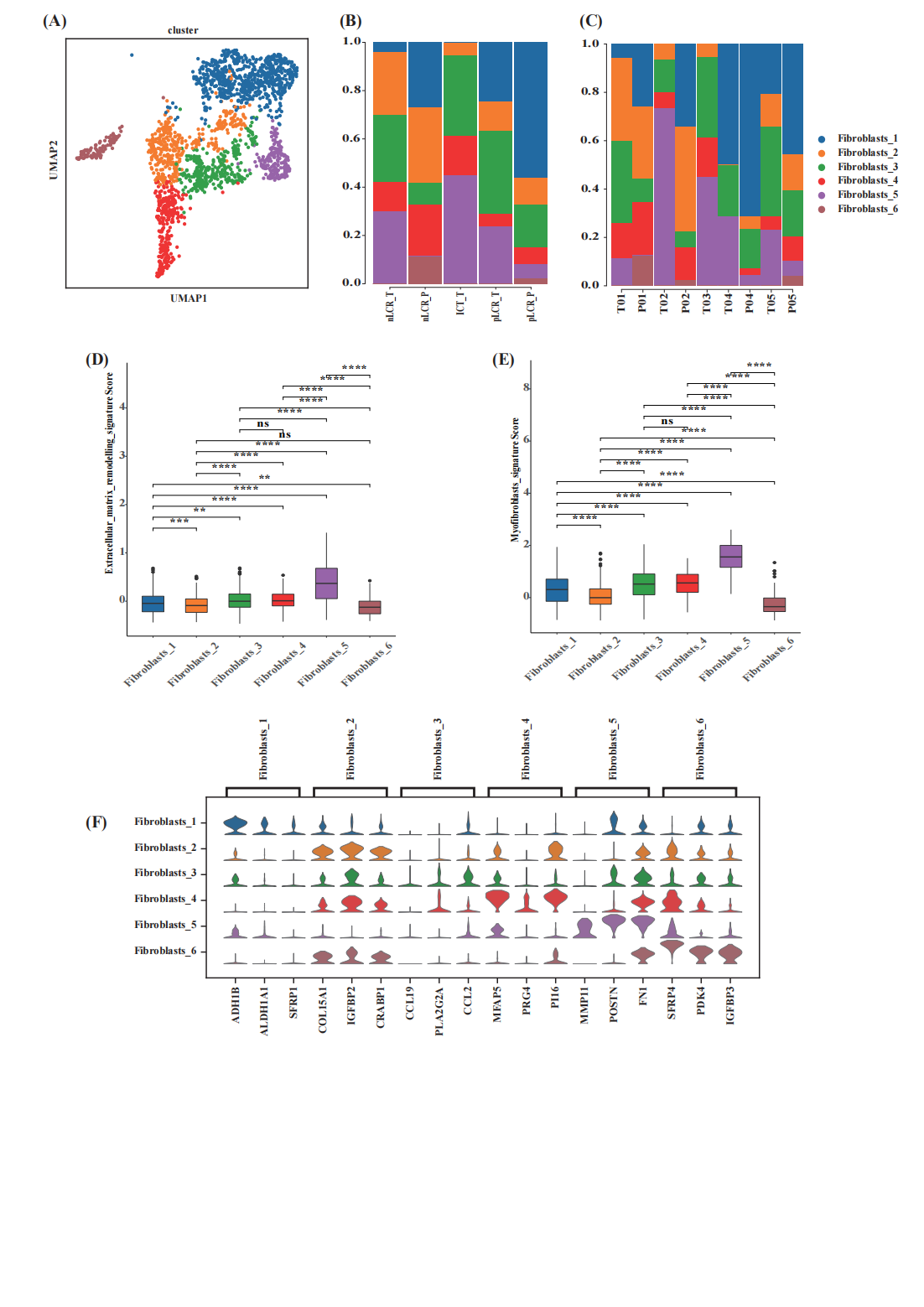


(A) UMAP plot of the fibroblast landscape, coloured by subcluster. (B, C) The proportions of fibroblast clusters by each sample subgroups and each sample. Samples were categorised based on the presence or absence of a pathological complete response in the axillary lymph nodes (pLCR and nLCR, respectively) and tissue type (tumour [T], paracancerous [P], and axillary lymph node [L]). One patient only had isolated tumour cells in the lymph node samples and was categorised separately (ITC). (D) Extracellular matrix remodelling signature scores of different subclusters of fibroblasts.(E) Myofibroblasts signature scores of different subclusters of fibroblasts. (F) Expression levels of typical marker genes in the fibroblast subclusters.

NAC, neoadjuvant chemotherapy; UMAP, uniform manifold approximation and projection.
